# Comparative phytochemical and biological evaluation of Egyptian *Swinglea glutinosa* stems and leaves

**DOI:** 10.1038/s41598-025-00427-2

**Published:** 2025-06-04

**Authors:** Reham Hassan Mekky, Mareena M. Thabet, Omayma El-Gindi, Mohamed Adel Said, Safwat A. Ahmed, Reda F. A. Abdelhameed

**Affiliations:** 1 Department of Pharmacognosy, Faculty of Pharmacy, Egyptian Russian University, Cairo-Suez Road, Badr City, 11829 Cairo Egypt; 2 Department of Pharmaceutical Chemistry, Faculty of Pharmacy, Egyptian Russian University, Badr City, 11829 Cairo Egypt; 3 Department of Pharmacognosy, Faculty of Pharmacy, Suez Canal University, Ismailia, 41522 Egypt; 4 Department of Pharmacognosy, Faculty of Pharmacy, Galala University, New Galala, 43713 Egypt

**Keywords:** *Swinglea glutinosa*, Rutaceae, 2-Deoxy-2,3-dehydro-*N*-acetyl-neuraminic acid, Alzheimer’s, Diabetes, Metabolomics, Chemical biology, Computational biology and bioinformatics

## Abstract

The hydroalcoholic extracts of both stems and leaves of Egyptian *Swinglea glutinosa* have been evaluated for their biological activities and phytochemical profiling. LC–MS/MS assists in identifying 80 phytoconstituent compounds that alternate between the stem and leaves, the majority of which are new to the genus. Biological investigation results revealed the superiority of stem extract in inhibiting α-amylase and α-glucosidase enzymes scoring IC_50_ (15.32 ± 0.76) and (0.656 ± 0.03) over the leaves extract, which gives IC_50_ (112.1 ± 5.55) and (2.721 ± 0.13) respectively at (P < 0.05) and when compared to the antidiabetic standard acarbose it shows better result than it in inhibiting α-amylase and to close to it in inhibiting α-glucosidase enzymes which later score IC_50_ (27.2 ± 1.35)and (0.375 ± 0.02) at (P < 0.05). Stem extract also shows good inhibitory activity on acetylcholinesterase enzymes compared to standard donepezil, and that was supported by results of intermolecular docking for six compounds (2-Deoxy-2,3-dehydro-*N*-acetyl-neuraminic acid (DANA), ascorbic acid, glucuronic acid, protocatechuic acid, galacturonic acid, gallic acid) which only identified in stem extract. All of them show high fitting scores and strong binding interactions. Moreover, among tested compounds, DANA, ascorbic acid, and glucuronic acid have the highest-ranking scores against all target enzymes. Highly fitting and binding scores by DANA were remarkably noticed, and the molecular dynamic simulation study proved the effect of DANA against the α-amylase enzyme via decreasing the fluctuations of the enzyme’s amino acid residues due to the stabilization of enzyme-ligand complex.

**Supplementary Information:**

The online version contains supplementary material available at 10.1038/s41598-025-00427-2.

## Introduction

Diabetes mellitus type two was and still is a chronic harsh disease that steals man’s life stepwise by affecting all functions of his biological organs if it remains uncontrolled^1–3^ Many researchers concluded that α-amylase and β-glucosidase are cornerstones of elevated glucose postprandial levels, so searching for enzyme inhibitors was essential in controlling this disease^4,5^. Alzheimer’s dementia (AD) is a century-old disease that many people are suffering from. All scientists studying AD agreed on correlating the effect of free radicals, primarily reactive oxygen species (ROS)^6^ and cholinesterase enzyme^7^ in its pathogenesis; hence, antioxidants and cholinesterase inhibitors became their targets in helping dementia people^8^.

Uncontrolled type 2 diabetes is one of the significant causes of cognitive impairment, which in turn accelerates the progression of Alzheimer’s disease, as evidenced by a multitude of published studies^5,9,10^.

Those investigations proposed different mechanisms for these relations, such as insulin resistance dysregulation leading to neuronal damage causing impaired cognition, another state that hyperphosphorylation of glycogen synthase kinase-3β, Aβ, tau may be the cause behind these relations and many more^9–13^.

Most patients prefer herbal medicine over synthetic drugs when its efficacy and safety-proven.

Consequently, investigating biological activities for herbs is still a wide field in which to spend effort. Diabetes and Alzheimer’s diseases were and still grabbing the attention of scientists due to the large number of people are suffering from them and the difficulties and challenges patients face every day from either the pathogenesis of the disease or the side effects of its medications^9,14^.

The Rue family (Rutaceae) contains about 158–160 genera, dividing approximately 1800–1900 species, most of which are woody shrubs and trees. Most plants belonging to Rutaceae are characterized by their flowers, which are generally showy and fragrant, and many species have attractive aromatic foliage^15^ Alkaloids, Cardiac glycosides, Coumarins, Flavonoids, Volatile oils, Tannins, and saponins were previously Reported in Rutaceae^11,16^.

Plants of the Rue family are reported to have many biological activities such as anti-inflammatory, analgesic, anti-lipidemia, anti-diabetic, anti-fungal, and anti-microbial, and many studies examined its anticancer activity against lung cancer, breast cancer, cervical cancer, and liver cancer and results were promising^17^. *Citrus* is the famous genus of this family as it contains essential food crops^18^, and upon that, Rutaceae is divided into *Citrus* plants and non-citrus plants.

*Swinglea glutinosa* (*S. glutinosa*) is a non-citrus Rutaceae plant that contains only one species (glutinous). Native to Southeast Asia and distributed to other countries, it is widely spread in Columbia to be used as a defense fence^19^. Few studies were conducted to investigate its active constituents, reporting the existence of acridone alkaloids in stem bark, roots, and leaves. Coumarins were detected in its roots. Severine alkaloids, essential oils, and Triterpenes were found in its fruits. Amides were isolated from its stems and fruits^20,21^. Antiparasitic, antioxidant, and antiproliferative activities were previously recorded for the plant^22^.

Our efforts were focused on investigating the 80% methanolic extracts of both leaves and stems of the *S. glutinosa* cultivated in Egypt chemically and biologically. The total phenolic and flavonoid contents of both leaves and stems methanolic extracts were measured. Additionally, their anticholinesterase, antidiabetic, and antioxidant properties were examined in vitro, and with the help of the precise technique of HPLC-ESI-QTOF-MS and tandem MS/MS analysis of hydroalcoholic extracts of both parts to investigate embraced phytoconstituents compounds. Then, dynamic molecular docking is used to correlate detected constituents with the results of in-vitro investigation.

## Materials and methods

### Chemical and reagents

#### Sample preparation and extraction procedure

Fresh leaves and stems were collected in June 2021 at Mazhar Botanic Gardens Baragil, Giza, Egypt. Mrs. Thérèse Labib, consultant of Plant Taxonomy at the Ministry of Agriculture and former consultant of El-Orman Botanic Garden, verified the samples. The voucher specimens with the identification number ERU S. GLUTINOSA_21 were stored at the Herbarium of the Department of Pharmacognosy, Faculty of Pharmacy, Egyptian Russian University.

50 g of powdered leaves were macerated in 80% methanol (0.5 L) and filtered. The previous steps were repeated four times. Finally, the extract was concentrated using a rotary evaporator, yielding 12 g of residue. The same steps were done with powdered stems, yielding 9.5 g of residue.

### Total phenolics and total flavonoid contents

Total phenolic content was quantified using the Folin–Ciocalteu technique, as published by^23,24^. Briefly, 250 µL of Folin–Ciocalteau reagent were added to the test tube with the extract (100 µL). Distilled water was added to make a total of 3.5 mL. After 5 min, 1.25 mL of a 20% aqueous sodium carbonate (Na_2_CO_3_) solution was added to neutralize the mixture. After 40 min, absorbance was measured at 725 nm compared to the solvent blank. A calibration curve of gallic acid was used to quantify total phenolic content in milligrams of gallic acid equivalent per gram of sample.

Flavonoid content was measured using an AlCl_3_ colorimetric approach, as described by^23^. In summary, 100 µL of extract was combined with 300 µL of 5% sodium nitrite solution. The technique involved adding a 10% AlCl_3_ solution after 6 min, followed by 300 µL of distilled water to reach a final volume of 2.5 mL. After adding 1.5 mL of 1 M NaOH, the sample was centrifuged at 5000*g* for 10 min. The supernatant absorbance was measured at 510 nm in relation to the solvent blank. A catechin calibration curve was used to quantify the overall flavonoid content in milligrams of catechin equivalent (mg CE) per gram of the sample.

### Antioxidant activity DPPH (2,2-diphenyl-1-picrylhydrazyl and ABTS (2,2′-azino-bis (3-ethylbenzothiazoline-6-sulfonic acid) assays

#### Determination of radical DPPH scavenging activity

The DPPH method was employed to evaluate the ability of extracts to scavenge free radicals as per^25^; the final concentration of DPPH was 200 µM, with a total volume of 3.0 mL. The absorbance was measured at 517 nm against a blank of purified methanol after 1 h of dark incubation. The percent inhibition of the DPPH free radical was determined using the following formula:

The inhibition (%) is calculated as 100 × [(Acontrol − Asample)/Acontrol].

The absorbance of the control reaction, which comprises all reagents except the test compound, functions as a control. The absorbance of the test substance functions as a sample.

The standard curve was prepared using Trolox. The results were expressed in Trolox equivalents (TE) per milligram (mg) of the sample.

#### Determination of radical ABTS scavenging activity

The stock solutions of the ABTS* reagent were prepared by reacting equivalent quantities of a 7 mM aqueous solution of ABTS* and 2.45 mM potassium persulfate in the dark at room temperature (25 °C) for 16 h in the dark, according to^25^. The working solution was subsequently prepared by diluting 1 mL of ABTS* solution with 60 mL of ethanol: water (50:50, v/v) to achieve an absorbance of 1.0 ± 0.02 units at 734 nm using a spectrophotometer (Spectro UV–VIS Double Beam, Model UVD-3500, Labomed, Inc. La Cienega Blvd. Los Angeles, CA 90034, USA). The extracts (50 µL) were allowed to react with 4.95 mL of the ABTS* solution in a dark environment for 1 h. The spectrophotometer was then employed to measure the absorbance at 734 nm. Trolox was employed to generate the standard curve. The results were reported in mg Trolox equivalents (TE) per gram of sample.

### In-vitro anti-diabetic activity (α-amylase and α-glucosidase inhibition assays) and acetylcholinesterase inhibition activity

#### α-Amylase

The assay mixture comprised 200 µL of 0.02 M sodium phosphate buffer, 20 µL of the enzyme, and plant extracts at a 20–100 µg/mL concentration. The test tubes were incubated at room temperature for 10 min, and then 200 µL of starch was added to each tube. The reaction was halted by adding 400 µL of 3,5-Dinitrosalicylic acid (DNS) reagent. After being maintained in a boiling water bath for 5 min, the mixture was chilled and diluted with 15 mL of distilled water, and the absorbance was measured at 540 nm. A control sample is a sample to which no plant extracts are added. The formula was employed to calculate the percentage of inhibition.

$${\text{Inhibition}}\;(\% ){\text{/Abs }}540\;({\text{control}}) = {\text{Abs}}\;540\;({\text{extract}}) \, - {\text{ Abs}}\;540\;({\text{control}}).$$

Using nonlinear regression analysis, the percent inhibition vs. log inhibitor concentration plots were employed to determine the values by calculating the half-maximal inhibitory concentration (IC_50_) values from the mean inhibitory values. Acarbose was the conventional alpha-amylase inhibitor employed. The assay was done in triplicates^26^.

#### α-Glucosidase

This assay was conducted according to^26^, where α-glucosidase was dissolved in 100 mM phosphate buffer at a pH of 6.8; the substrate was *P*-nitrophenyl-α-D-glucopyranoside. The extracts were added at concentrations between 20 and 100 µg/mL. Five minutes at 30 °C were spent mixing 320 µL of 100 mM phosphate buffer pH 6.8 with varying concentrations of plant extracts. After adding 3 mL of 50 mM sodium hydroxide to the mixture, the absorbance at 410 nm was measured. Plant extracts were not used in the preparation of the control samples. The following formula was used to compute the percentage inhibition.

$${\text{Inhibition}}\;(\% ) = \frac{{{\text{Abs}}\; 410 \;({\text{control}}){-} {\text{Abs }}\;410\;({\text{extract}})*100}}{{{\text{Abs}}\;410\;({\text{control}})}}$$

The IC_50_ values were computed using nonlinear regression analysis based on the mean inhibitory values and were obtained from plots of percent inhibition versus log inhibitor concentration. The standard α-glucosidase inhibitor was acarbose. Every test was run in triplicate.

#### Acetylcholinesterase inhibition assay

The modified approach was utilized to determine the enzymatic activity of acetylcholinesterase (AChE)^27^. In a 96-well microplate, 25 mL of 15 mM ATCI in MilliQwater, 125 mL of 3 mM DTNB in buffer C (50 mM Tris–HCl, pH 8, 0.1 M NaCl, 0.02 M MgCl_2_·6H_2_O), and 25 mL of the sample were combined with 50 mL of buffer (50 mM Tris–HCl, pH 8, 0.1% bovine serum albumin). The absorbance was continuously monitored at 415 nm for 2 min at 5-s intervals using a Sunrise microplate reader (P-Intertrade Equipment, Australia) after adding 25 mL of 0.22 U/mL of AChE. The enzyme activity was determined by comparing the reaction rates between the samples and the control (25 mL of 10% methanol in a buffer, which was used in place of the sample). The proportion of enzyme activity was subtracted from 100% to ascertain the percentage of inhibitory activity. The experiment was conducted three times. Donepezil was employed as the standard medication, while a buffer solution was used as the negative control. The extract concentration that elicited 50% inhibition (IC_50_) was determined, and the percentage inhibition was calculated. The following is the calculation of the percentage inhibition (I%):

$$\begin{aligned} & \% \;{\text{Relative}}\;{\text{Inhibition}} = \left( {\left( {{\text{Slope}}\; \, \left[ {\text{S}} \right] - {\text{Slope}}\;{\text{of}}\;\left[ {\text{S}} \right]} \right)/{\text{Slope}}\;{\text{of}}\;\left[ {{\text{EC}}} \right]} \right)*100 \\ & \% {\text{ Relative}}\;{\text{Activity}} = \left( {{\text{Slope}}\;{\text{of}}\;\left[ {\text{S}} \right]/{\text{Slope}}\;{\text{of}}\;\left[ {{\text{EC}}} \right]} \right){\text{ X - }}100. \\ \end{aligned}$$

### RP-HPLC-ESI-QTOF-MS and tandem MS/MS

Chromatographic separations were performed on a Poroshell 120 HiLiC Plus column (150 mm 3 mm, 2.7 μm particle size, Agilent Technologies, Santa Clara, CA, USA) using an Agilent 1200 series rapid resolution system (Agilent Technologies). The system was equipped with a quaternary pump (G7104C) and an autosampler (G7129A)^28,29^. The mobile phases for gradient elution were acetonitrile (phase B) and acidified water (0.5% acetic acid, v/v) at a 0.2 mL/min flow rate. The replicates of the extracts were analyzed, and the injection volume was 5 µL. The gradient elimination was as follows: 0 min (99% A, 1% B), 5.50 min (93% A, 7% B), 11 min (86% A, 14% B), 17.50 min (76% A, 24% B), 22.50 min (60% A, 40 B), 27.50 min (0% A, 100% B), 28.50 min (0% A, 100% B), 29.50 min (99% A, 1% B), and 35 min (99% A, 1% B). The following operating conditions were briefly described: a drying nitrogen gas temperature of 325 °C with a flow rate of 10 L/min, a nebuliser pressure of 20 psig, a sheath gas temperature of 400 °C with a flow rate of 12 L/min, a capillary voltage of 4000 V, a nozzle voltage of 500 V, a fragmentor voltage of 130 V, a skimmer voltage of 45 V, and an octapole radiofrequency voltage of 750 V. MassHunter Workstation software (Agilent Technologies) was used to regulate data acquisition in profile mode at a rate of 2.5 Hz. Over a mass-to-charge (m/z) range of 70 to 1100, the spectra were acquired in negative-ion mode. The detection window was established at 100 ppm^30–32^.

### Intermolecular docking

The molecular operating environment (MOE) 2019.0102 was utilized for the synthesis of proteins and ligands, molecular docking, and assessment of ligand–protein interaction via pose visualization and scoring function.

#### Simulations of molecular docking

For the synthesis of proteins and ligands, molecular docking, and assessment of ligand–protein interaction via pose visualization and scoring function, the molecular operating environment (MOE) 2019.0102 was utilized. Using the MMFF94x protocol, the docking operation was carried out using docking placement: Triangle Matcher, London dG for rescoring, Forcefield, and Affinity dG for refining^33,34^.

#### Preparation of target protein structure

Α-amylase enzyme [PDB id: 1b2y], α-glucosidase enzyme [PDB id: 3wy2], and acetylcholine esterase ACHE enzyme [PDB id: 6tt0] were obtained from http://www.rcsb.org, the Protein Data Bank. Because of their crucial role in anti-diabetic and anti-Alzheimer drug design through evolving research^35^. The protein structure was examined and fixed using MOE’s automatic correction and fixing order. During the protonation process, hydrogen atoms were added to the structure.

The co-crystalized ligands were used to determine the binding sites. Then, the co-crystallized water molecules and bound ligands were removed. After docking completion, results were observed and filtered through scoring values and visualized poses.

#### Preparation of tested drug molecules

MOE was utilized to create the 3D model library of target compounds. These compounds were subjected to an energy minimization process and the automatic calculation of the partial charges. Finally, this prepared library was saved as a Microsoft Database (MDB) file for the docking calculations.

#### Validation of docking

The root mean square deviation (RMSD) is calculated to validate the docking process. Redocking the co-crystallized ligand on its target enzyme and then superimposing it on its initial co-crystallized constrained conformation allows for the prediction of the RMSD.

During this study, RMSD values of 1.8639, 1.4023, and 1.6685 Å for α0-amylase [PDB id: 1b2y], α-glucosidase [PDB id: 3wy2], and acetylcholine esterase ACHE [PDB id: 6tt0] enzymes were found to be within the acceptable range.

#### Molecular dynamic simulations

Based on the docking results and biological activity, the highest-ranked and best-posed docked complex was subjected to a molecular dynamic simulation study. The MD simulations were carried out by the cabs-flex 2.0 server (http://biocomp.chem.uw.edu.pl/CABSflex2/). Molecular dynamics simulations were performed using the CABS Flex 2.0 server and were based on the coarse-grained simulations of protein motion^36^. Over 50 cycles and 50 trajectory frames of 10 ns.

MMFF94x protocol was used to carry out the docking process, and docking placement was used: Rescoring: London dG, Forcefield and refinement: Affinity dG, triangular matcher^33,34^.

### Statistical analysis

GraphPad Prism (Version 10) was used to conduct the analyses. The data were presented as the mean ± standard deviation (SD). A one-way ANOVA was implemented, followed by Tucky post-hoc analysis. P < 0.05 was established as the statistically significant level in all tests.

Minitab 17 (Minitab, Inc., USA) was employed for bubble graphs, while Microsoft Excel 365 (Redmond, WA, USA) was employed for conditional formatting.

## Results and discussion

### Total phenolics and total flavonoid contents

The phenolic contents of stems and leaves were close to each other, scoring 29.371 mg GAE and 32.008 mg GAE, while the flavonoid contents of leaves were 8.059 mg CE, which was nearly double that of stems, which scored 4.688 (mg CE) (*p* < 0.05) (Table 1).

**Table 1:** Total phenol contents, total flavonoids contents, antioxidant activity, of the stems and leaves extracts of *Swinglea glutinosa.*

Samples	Activity			
	Total phenols (mg GAE/g)	Total flavonoids (mg CE/g)	DPPH (mg TE/g)	ABTS (mg TE/g)
Stems	29.371 ± 0.025***	4.688 ± 0.044***	8.62 ± 0.03***	23.295 ± 0.017***
Leaves	32.008 ± 0.023***	8.059 ± 0.024***	7.92 ± 0.051***	21.602 ± 0.036***

Results represented as mean ± SD (measuring in triplicate n = 3) using paired *t*-test. *** means significant (p < 0.0005).

### Antioxidant, antidiabetic and acetylcholinesterase inhibition activities

The inhibitory activity of stem extract appears to exceed about 10 times that of leaves and is much closer to that of standard donepezil with IC_50_ 2.36 ± 0.12, 30.72 ± 1.57, and 0.329 ± 0.02 μg/mL, respectively (Tables 1, 2). Many rue plants are reported to have neuroprotective activity either due to their antioxidant role or inhibitory activity of acetylcholinesterase enzyme, or a combination of both for *Citrus hystrix* plant fruit peel and fresh juice were proven to be potent antioxidants, and acetylcholinesterase inhibitor^37^. Also, leaves and peels of *Citrus aurantifolia* was reported to possess strong antioxidant and cholinesterase activity, which is attributed to their phytochemical content, mainly apigenin, which is abundant in our plant in glycoside forms. Continuing with the *C. aurantifolia* plant in Thailand after hydrodistillation of its fresh leaves and obtaining its essential oils, it also recorded promising cholinesterase inhibitory activity when compared to the galantamine standard^38^ by addressing the essential oil of the citrus family and its great biological value in a previously reported study, mice hippocampus were treated with the essential oil of *Citrus sinensis* for 30 consecutive days at 3 different doses. The inhibition of acetylcholinesterase esterase level was markedly noticed with all treated doses^39^. In Poland, when evaluating acetylcholinesterase and butyrylcholinesterase inhibitory activity of different European herbs, the hexane fraction of *Ruta graveolens* L. at 400 mg mL^−1^ got the second rank among all studied plants^40^.

**Table 2:** α-glucosidase, α-amylase and acetylcholine esterase activity of the stems and leaves extracts of *Swinglea glutinosa.*

Samples	Activity		
	α-Glucosidase (IC_50_)	α-Amylase (IC_50_)	ACHE (IC_50_)
Stems	0.656 ± 0.03*^,^****	15.32 ± 0.76**^,^****	2.36 ± 0.12^ns,^****
Leaves	2.721 ± 0.13**** ****	112.1 ± 5.55**** ****	30.72 ± 1.57**** ****
Acarbose	0.375 ± 0.02* ****	27.2 ± 1.35** ****	
Donepezil			0.329 ± 0.02^ns,^****

Results represented as mean ± SD (measuring in triplicate n = 3) using ANOVA followed by post-hoc test. *ns* non-significant, * means significant (p < 0.05), ** means significant (p < 0.005), **** means significant (p < 0.0001).

The result of α-amylase was very impressive as the IC_50_ of stem extract was 15.32 ± 0.76 μg/mL which is less than that of the standard acarbose whose IC_50_ is 27.2 ± 1.35 μg/mL at (*p* < 0.05)while leaves were of the least inhibitory potential by scoring the highest IC_50_ 112 ± 5.55 μg/mL and same results found with testing α-glucosidase inhibitory activity the stem extract found to be much more potent recording IC_50_ (0.656 ± 0.03) μg/mL lower than that of leaves (2.721 ± 0.13) μg/mL and more close to the standard α-glucosidase inhibitor acarbose (0.375 ± 0.02) μg/mL at (*p* < 0.05). *Feronia limonia* plant of the same subtribe of our plants Balsamocitrinae, previously recorded significant antidiabetic activity through α-amylase and α-glucosidase inhibitor pathways which its aqueous and methanolic fruit extracts were tested against standard drug acarbose and results were promising^41^ also, isolated compounds from roots of *Paramignya trimera* of the same tribe citreae were superior to standard acarbose in alpha-glucosidase inhibition^42^. In Morocco, a study was conducted to evaluate the activity of different extracts of *Citrus aurantium* (L) Peels on alpha-amylase and alpha-glucosidase enzymes. It was promising to find that at dose 332 g/mL, all extracts inhibit all target enzymes by nearly 98% exceeding standard acarbose and in same study results of correlation analysis attribute the α-amylase inhibition to gallic acids contents which will state later on its presence in stem extract only of Egyptian *Swinglea glutinosa.*

The inhibitory activity of Stem extract appears to exceed about 10 times that of leaves and is much closer to that of standard donepezil with IC_50_ 2.36 ± 0.12, 30.72 ± 1.57, and 0.329 ± 0.02 μg/mL at (*p* < 0.05) respectively. Many rue plants are reported to have neuroprotective activity either due to their antioxidant role or inhibitory activity of acetylcholinesterase enzyme or a combination of both for the *C. hystrix* plant, whose fruit peel and fresh juice were proven to be potent antioxidants, and acetylcholinesterase inhibitor^37^ also leaves and peels of *C. aurantifolia* was reported to possess strong antioxidant and cholinesterase activity, which is attributed to their phytochemical content, mainly apigenin, which is abundant in our plant in glycoside forms. Continuing with *C. aurantifolia* plant in Thailand after hydrodistillation of its fresh leaves and obtaining its essential oils, it also recorded promising cholinesterase inhibitory activity when compared to the galantamine standard^38^ by addressing essential oil of citrus family and its great biological value in previously reported study mice hippocampus were treated with essential oil of *Citrus sinensis* for 30 consecutive days at 3 different doses. It was markedly noticed the inhibition of acetylcholinesterase esterase level with all treated doses^39^*.* In Poland, when evaluating acetylcholinesterase and butyrylcholinesterase inhibitory activity of different European herbs, the hexane fraction of *Ruta graveolens L*. at 400 mg mL^−1^ got the second rank among all studied plants^40^.

### RP-HPLC-ESI-QTOF-MS and tandem MS/MS analysis

**Fig. 1:**
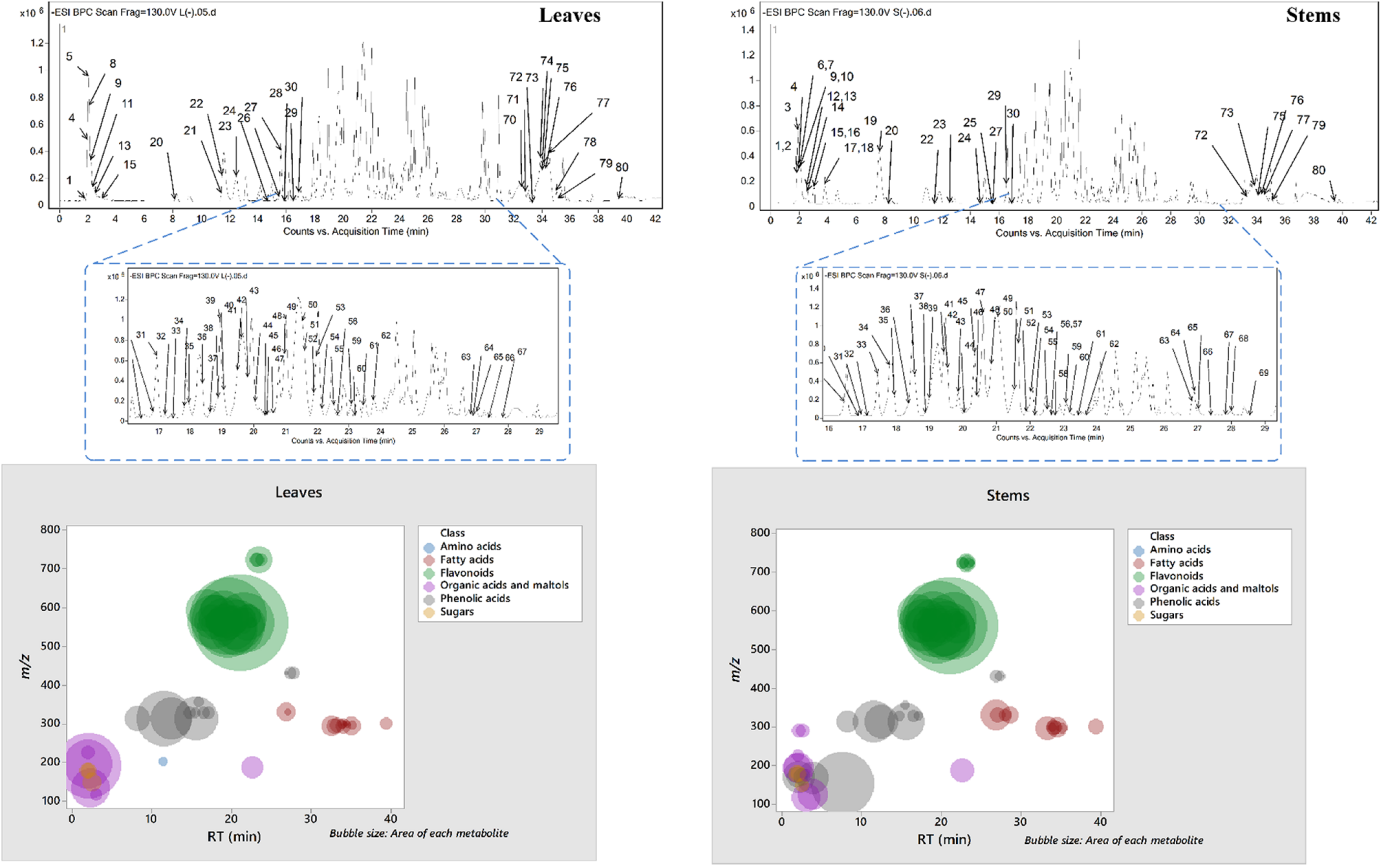
Base peak chromatograms (BPCs) of leaves and stems of *Swinglea glutinosa*, bubble plot of the identified masses *m/z vs* corresponding retention time concerning metabolites classes and peak areas for the stems and leaves.

**Fig. 2:**
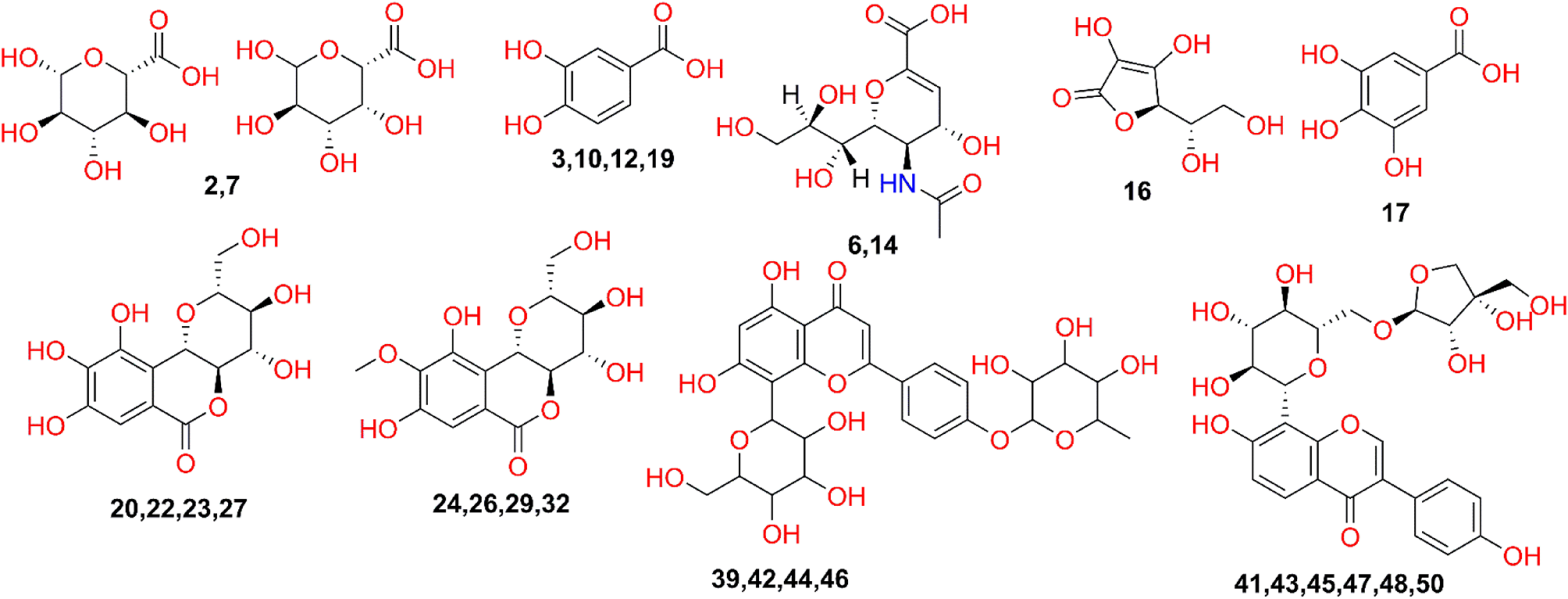
Structures of the main identified metabolites in the leaves and stems.

The metabolic profiling of the leaves and stems of *Swinglea glutinosa* unraveled the occurrence of 80 metabolites being classified as phenolic (49) and non-phenolic (31) compounds. In this context, the characterized phenolics belonged to flavonoids and phenolic acids, and the non-phenolics were subgrouped into organic acids, amino acids, sugars, and fatty acids. Figure 1 demonstrates the leaves and stems’ base peak chromatogram (BPC) and exhibits the bubble plot of the identified masses (*m/z*) plotted against the corresponding retention times for different metabolite classes, along with the peak areas for the leaves and stems^43,44^. Besides, Fig. 2 portrays the structures of the main bioactive identified metabolites in the leaves and stems.

#### Phenolic compounds

Most identified metabolites belong to this class. 31 flavonoids were detected, 19 of them belonging to flavones, 2 flavanones, and 10 isoflavones.

##### I.I.a Flavones

Peak 30 at Retention time (Tr) 16.86 min corresponds to the [M–H]^−^ at *m*/*z* 593.1493 with molecular formula C_27_H_30_O_15_. Its MS/MS spectrum shows that glycosyl flavone fragmentation is characterized by fragment ions at *m/z* 353 (Aglycone + 83) and 383 (Aglycone + 113)^45^, suggesting the tri-hydroxylated nature of the aglycone (apigenin, MW 270), providing additional evidence of di-*C*-glycosyl apigenin. The ion at *m/z* 503 [M−H−90]^−^ could be produced by loss of *C*-hexoside or pentoside but molecular weight confirming hexoside also The ion at *m/z* 473 [M−H−120]^−^ which indicates loss of *C*-hexoside so the compound was tentatively identified as Apigenin di-*C*-hexoside which is first time to be reported in the *Swinglea* genus but previously reported in Rutaceae family as Vicenin-2^46^ (Table 3).

**Table 3:**
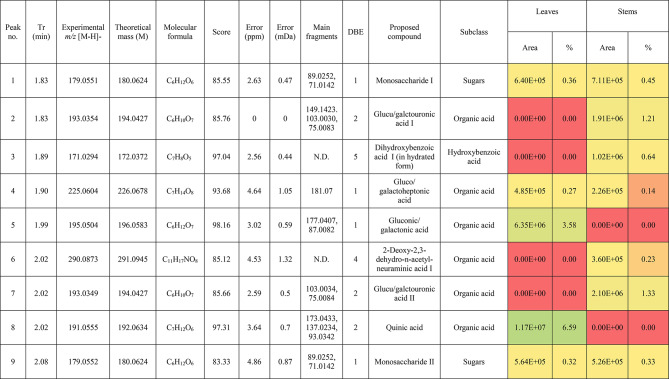

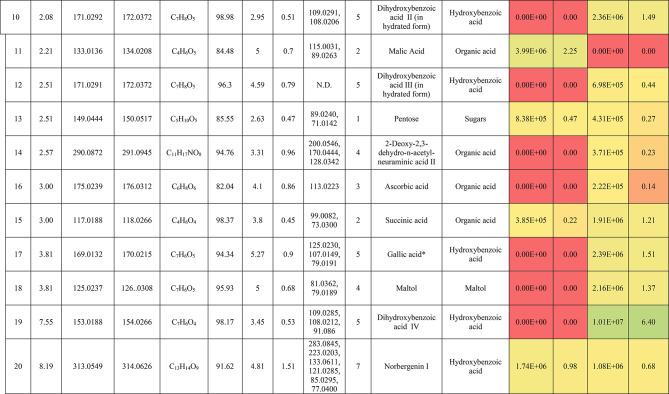

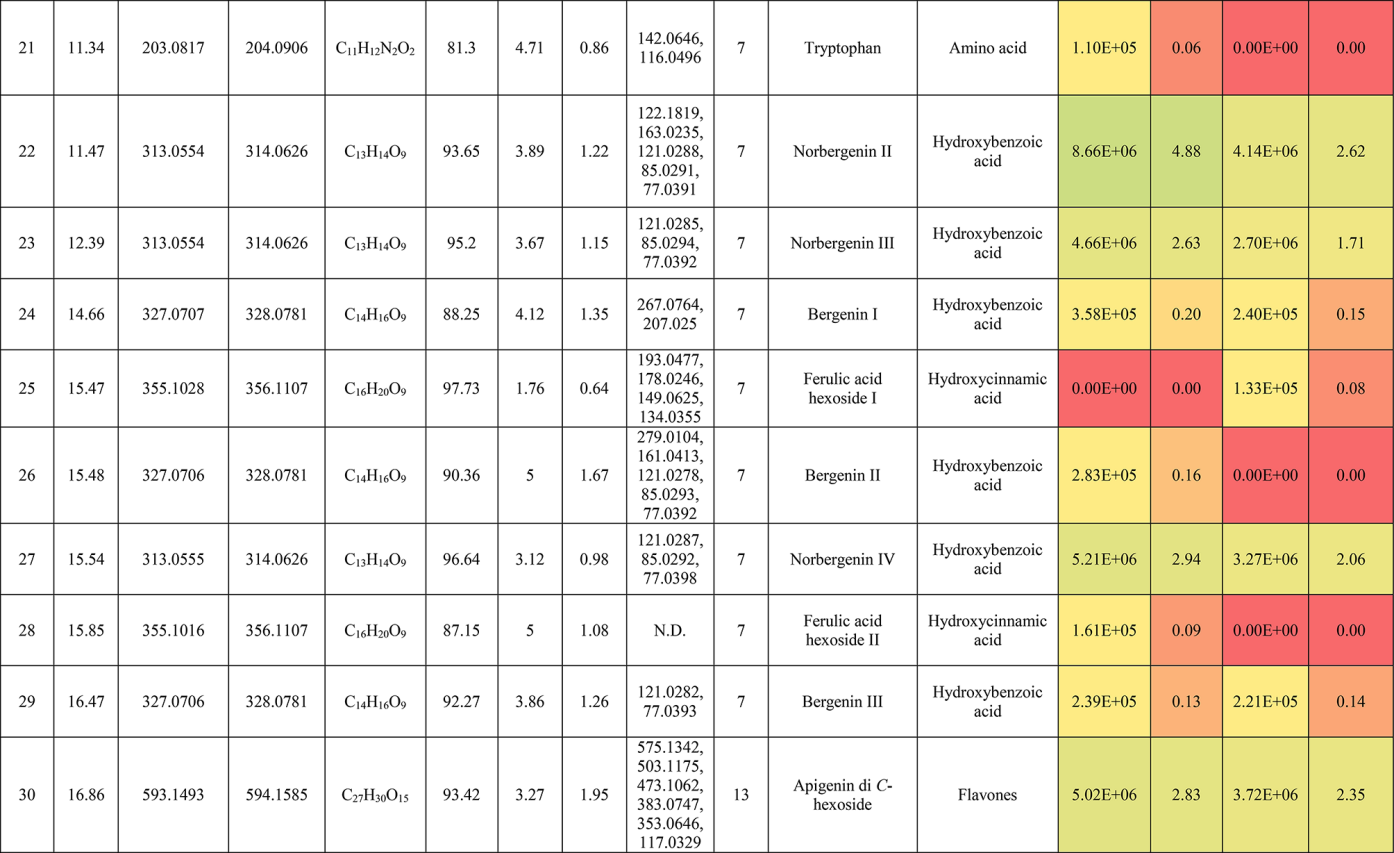

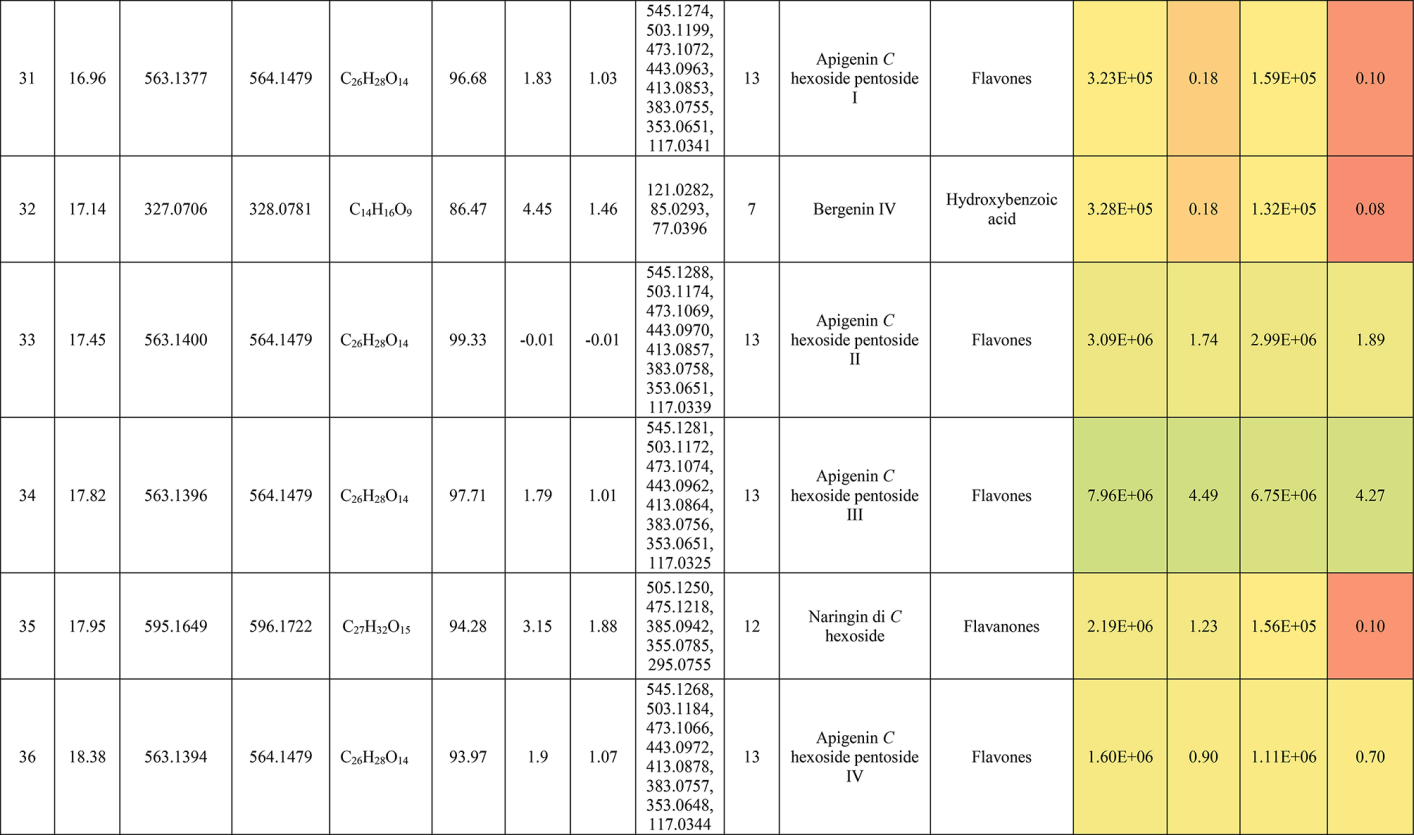

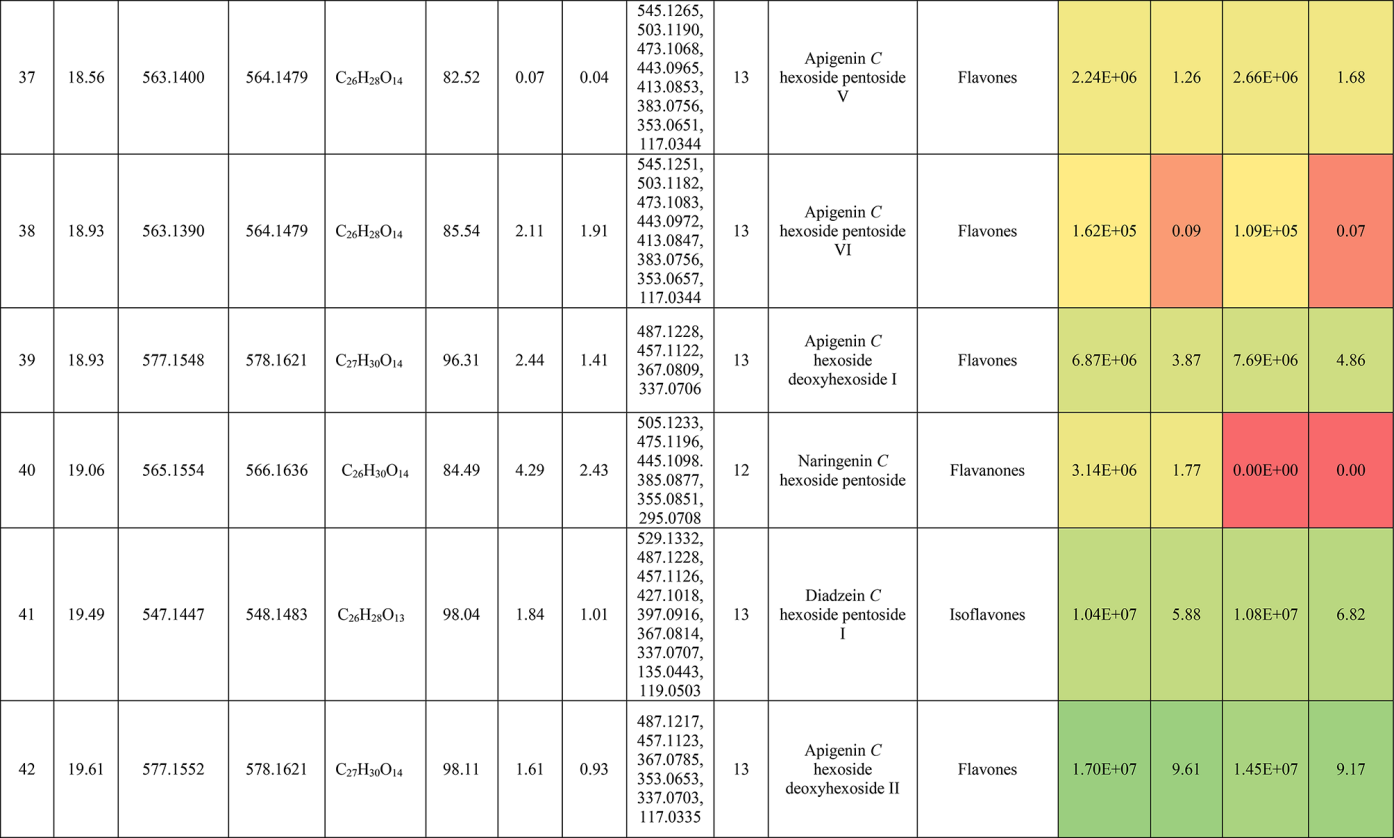

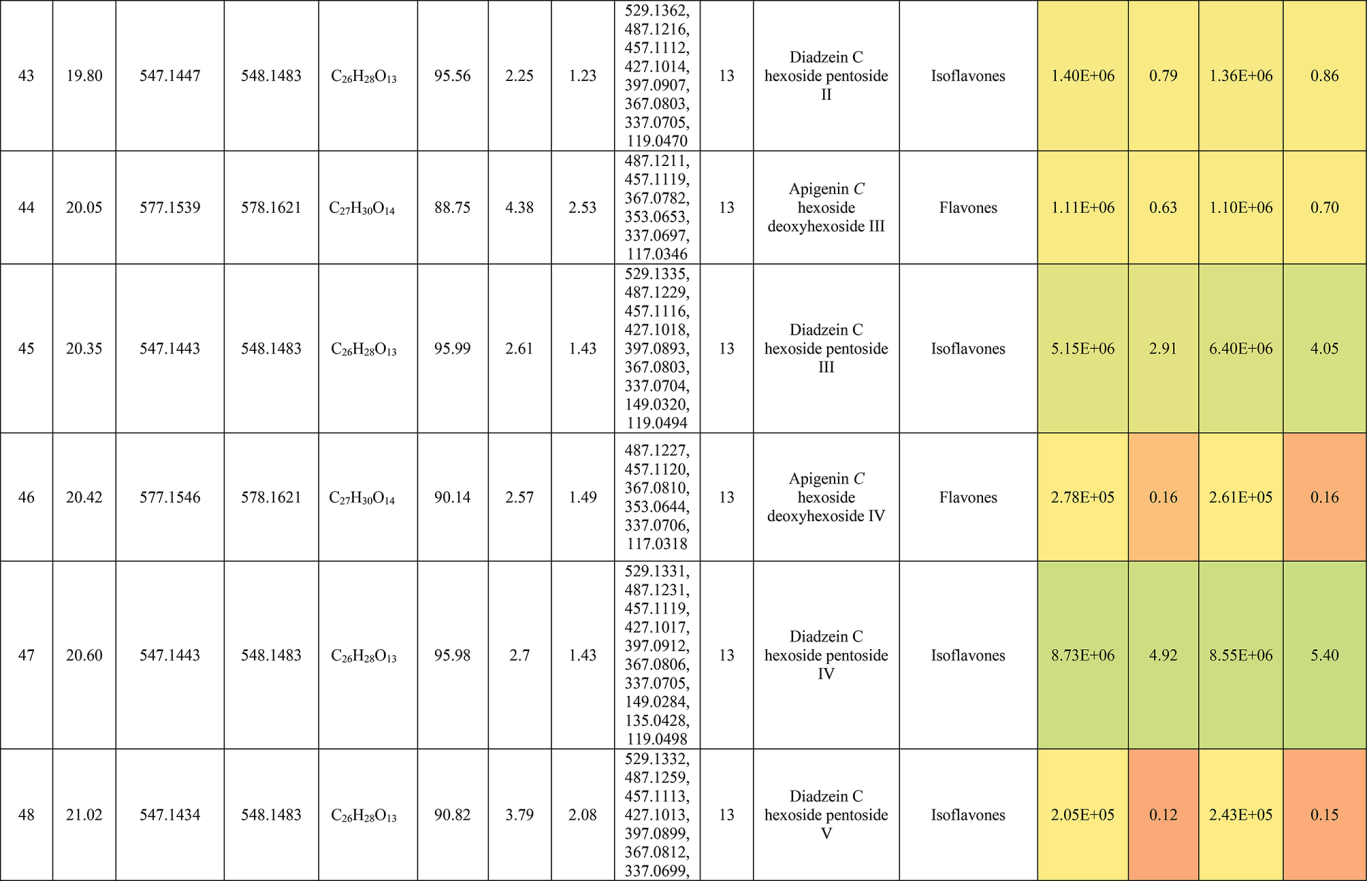

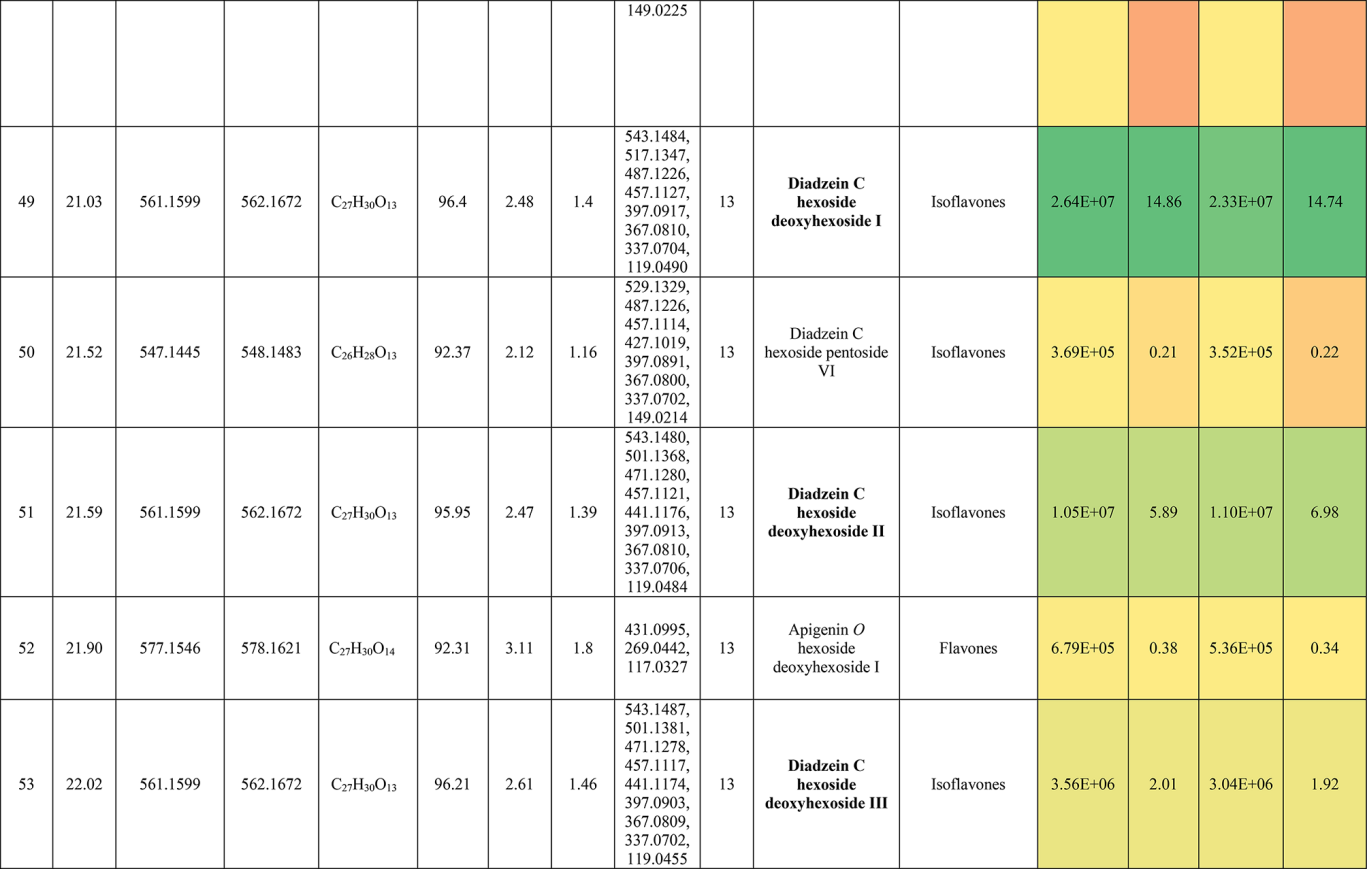

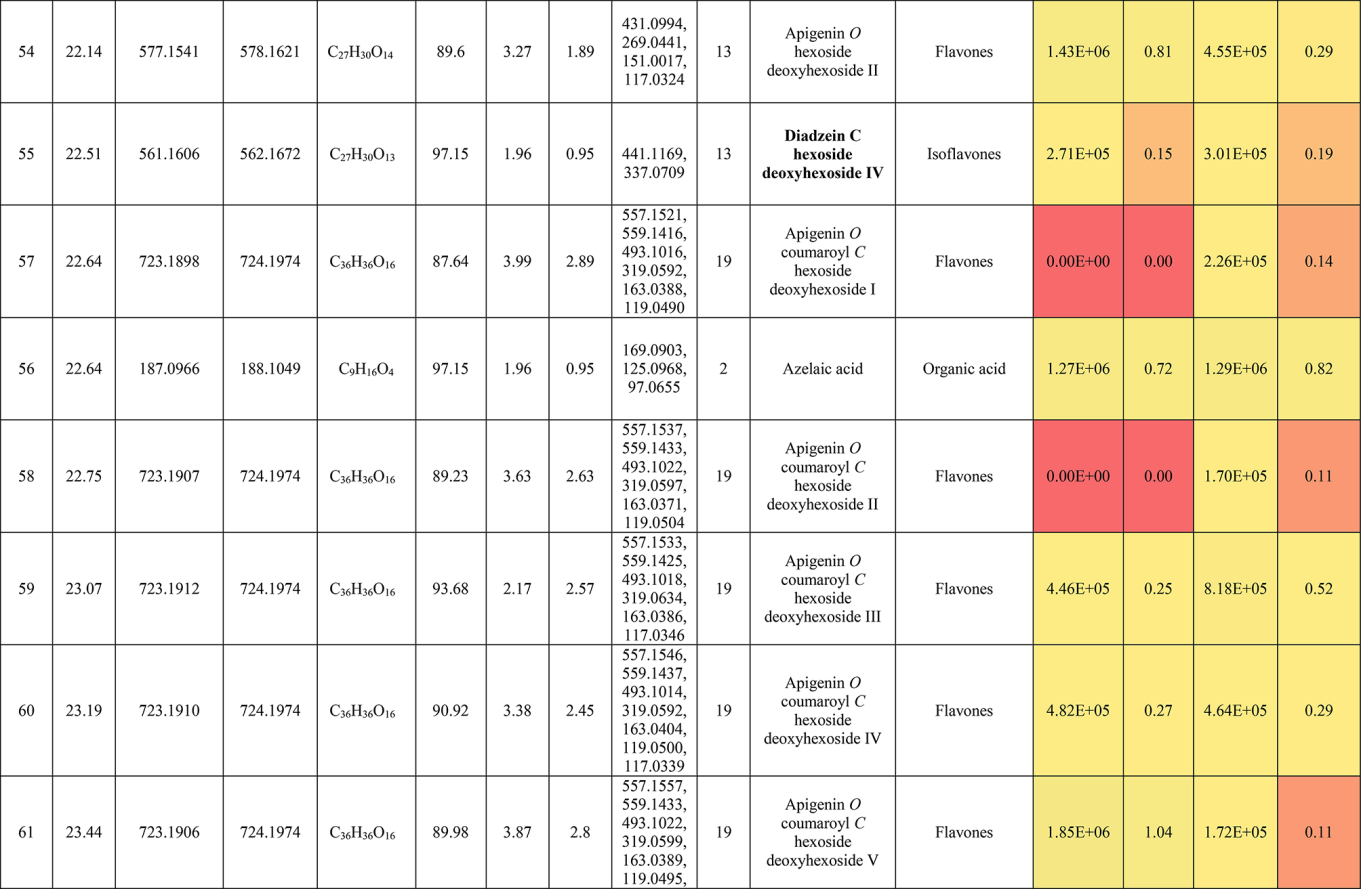

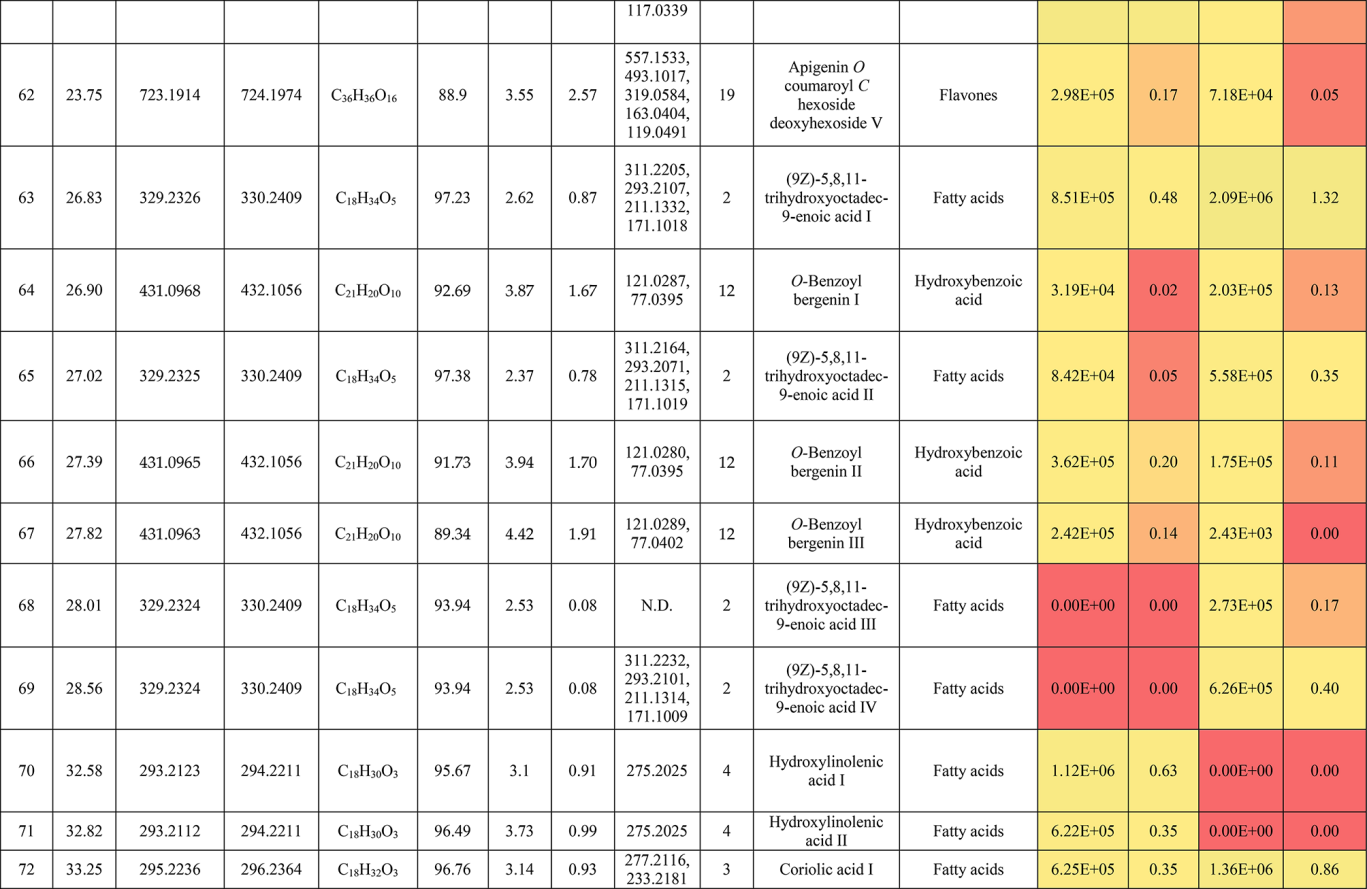

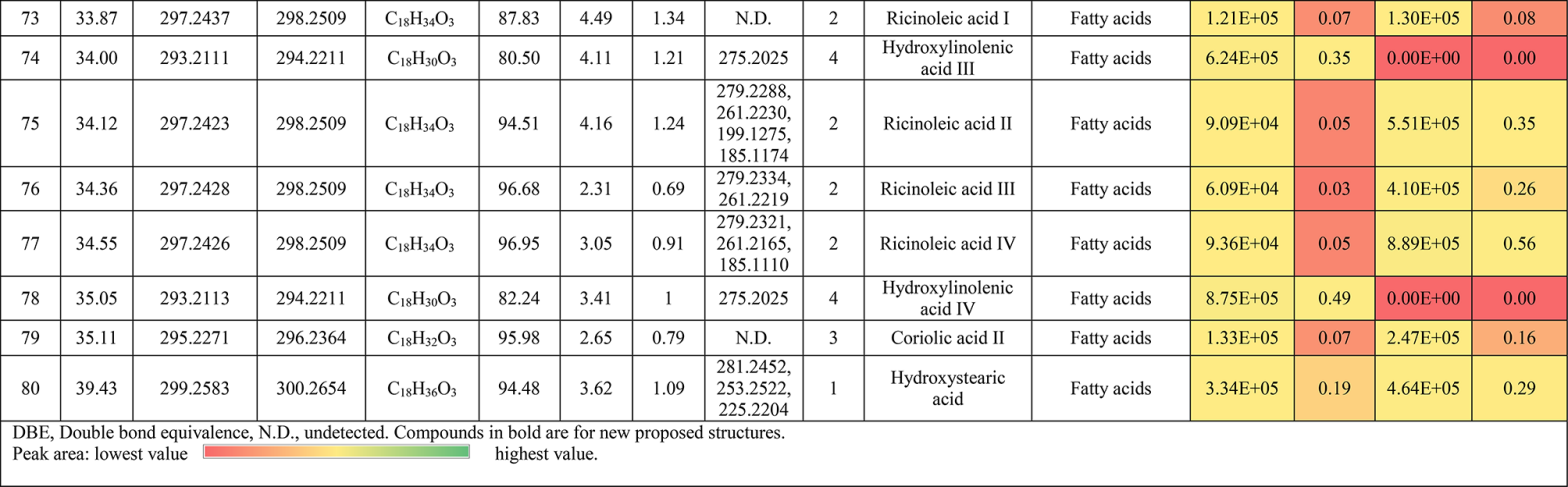
Metabolites characterized in the leaves and stems of *Swinglea glutinosa.*

**Fig. 3:**
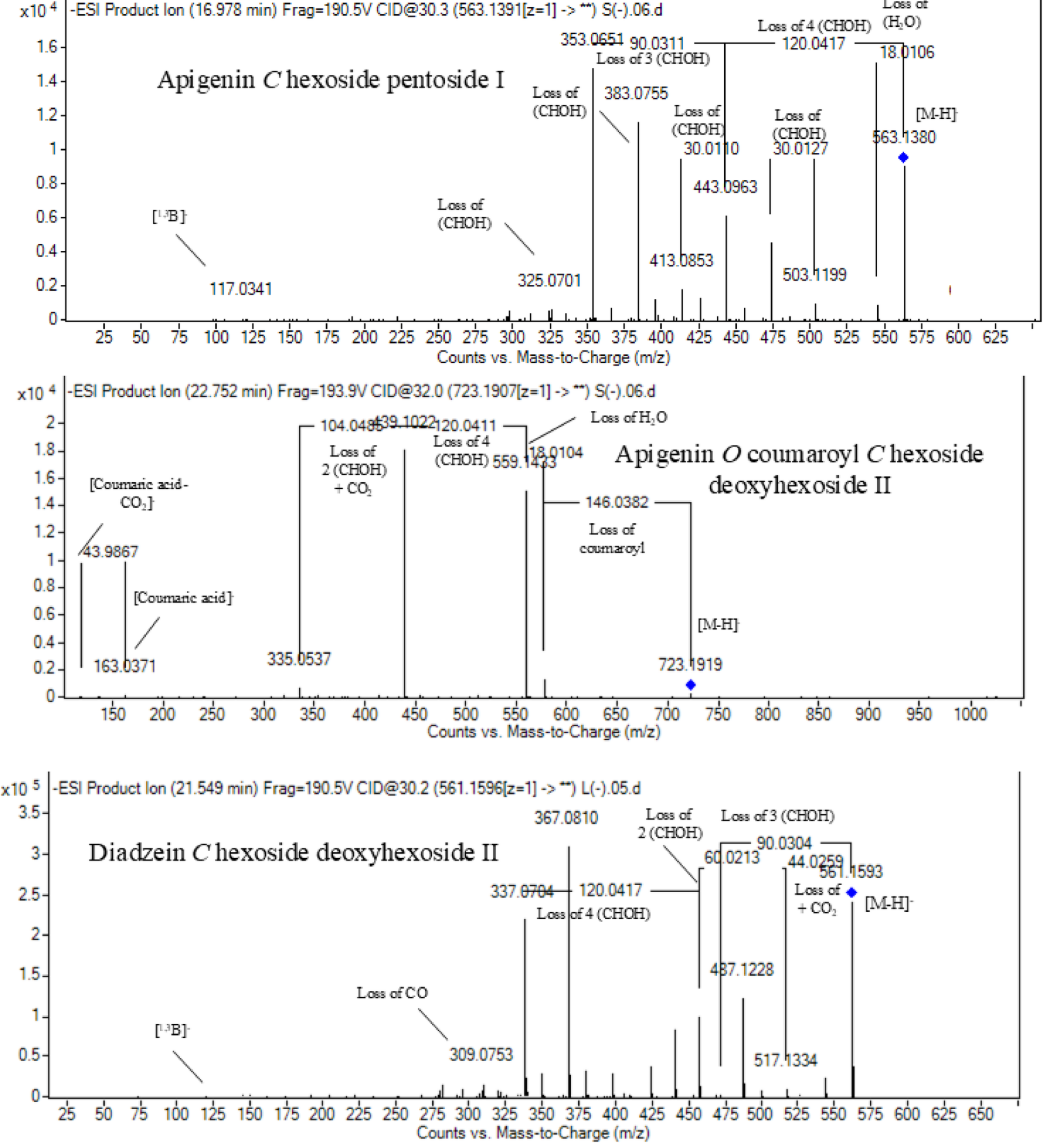
Pattern of fragmentation of apigenin *C* hexoside pentoside I, apigenin *O* coumaroyl *C* hexoside deoxyhexoside II, diadzein *C* hexoside deoxyhexoside II.

At the same pattern of fragmentation, Peak no. 31 at RT 16.96 min corresponds to [M–H]^−^ at *m*/*z* 563.1493 with molecular formula C_27_H_30_O_15_ its MS/MS spectrum shows fragment ions at *m/z* 353 (aglycone+83) and 383 (aglycone+113), which are indicative of the di-*C*-glycosyl flavone fragmentation in our case was apigenin^45^, The ion corresponds to *m/z* 503 [M−H−90]^−^ may be produced by loss of c-hexoside or pentoside but molecular weight Verifying pentoside also The ion at *m/z* 473 [M−H−120]^−^ which indicate loss of *C*-hexoside so the compound was tentatively identified as Apigenin-*C*-hexoside pentoside I (Fig. 3) with its 5 isomers at peaks 33,34,36,37and 38^47,48^ to our knowledge its first time to be detected in the Rue family but was previously reported in family Theaceae as Apigenin 6-*C*-glucosyl-8-arabinoside and Apigenin-6-*C*-arabinosyl-8-*C*-glucoside^49^.

As the aforementioned sequence of fragmentation of *C*-glycosyl flavone fragmentation peak no 39,42,44,46 with [M–H]^−^ at *m*/*z* 577.1548 was identified as Apigenin *C* hexoside deoxyhexoside isomers I–IV^50^ was previously reported in the family Rutaceae as Vitexin rhamnoside^51^ (Table 3).

Peak 52 and 54 at RT 20.42, 21.90 min corresponding to the [M–H]^−^ at *m*/*z* 577.1546. In an analysis of the MS/MS spectrum, predominant fragments at *m*/*z* 270 [M–H–308] were observed referring to the loss of 308 Da (162 + 146), suggesting that a hexose and a deoxyhexose are attached at the same position of the aglycone. This is verified by the presence of the resulting ion at *m/z* 431, which is attributed to the neutral loss of a deoxyhexose moiety (C_6_H_10_O_4_, calculated 146.0579 Da). Thus, the Compound was tentatively identified as Apigenin *O* hexoside deoxyhexoside isomers I, II and it was reported before in the family Rutaceae^52^ but newly in the *Swinglea* genus.

Peak no 57 at RT 22.64 min with [M–H]^−^ at *m*/*z* 723.1898 with predominant fragments at 559.1416 corresponding to *O* coumaryl moiety (146.0368 Da) loss followed by water [M–H-146-18] and 493.1022 [M–H-120-104] referring to loss of *C*-hexose and deoxyhexose. Thus, it was identified as Apigenin *O* coumaroyl *C* hexoside deoxyhexoside (Fig. 3) with the same pattern peaks from 58–62, which were identified as its isomers^53^. Vitexin has previously been reported in the Rutaceae family, but to our knowledge, this is the first time that its coumaroyl combination has been detected in the Rutaceae family (Table 3).

##### I.I.b. Flavanones

Peak no 35 compounds were identified as naringenin *C*-di-hexoside which previously detected in the family as Naringenin-6,8-di-*C*-Glucoside^54^ and peak 40 was identified as naringenin *C*-hexoside-pentoside exhibiting continued loss of 30 Da gives us a clear clue of the c-fragmentation pattern^55^ (Table 3).

##### I.I.c. Isoflavones

Six peaks with *m*/*z* 547.15 showed the neutral loss of H_2_O then successive loss of 60 Da and 30 Da which is typical for *C* glycosylation the compound was tentatively identified as Diadzein *C* hexoside Pentoside and detected before in family Fabaceae as mifricin^56^. To our knowledge, it is the first time it has been detected in the family Rutaceae. With the same pattern, four peaks *m/z* 561.16 and molecular formula C_27_H_30_O_13_ portrayed a *C*-glycosylation fragmentation pattern with the ion of m/z 119.05 characteristic for the ion [1,3B]- of daidzein fragmentation, and hence they were identified as Diadzein *C* hexoside deoxyhexoside I-IV (Fig. 3, Table 3).

### II. Phenolic acids

#### II.I. Hydroxybenzoic acids

**Fig. 4:**
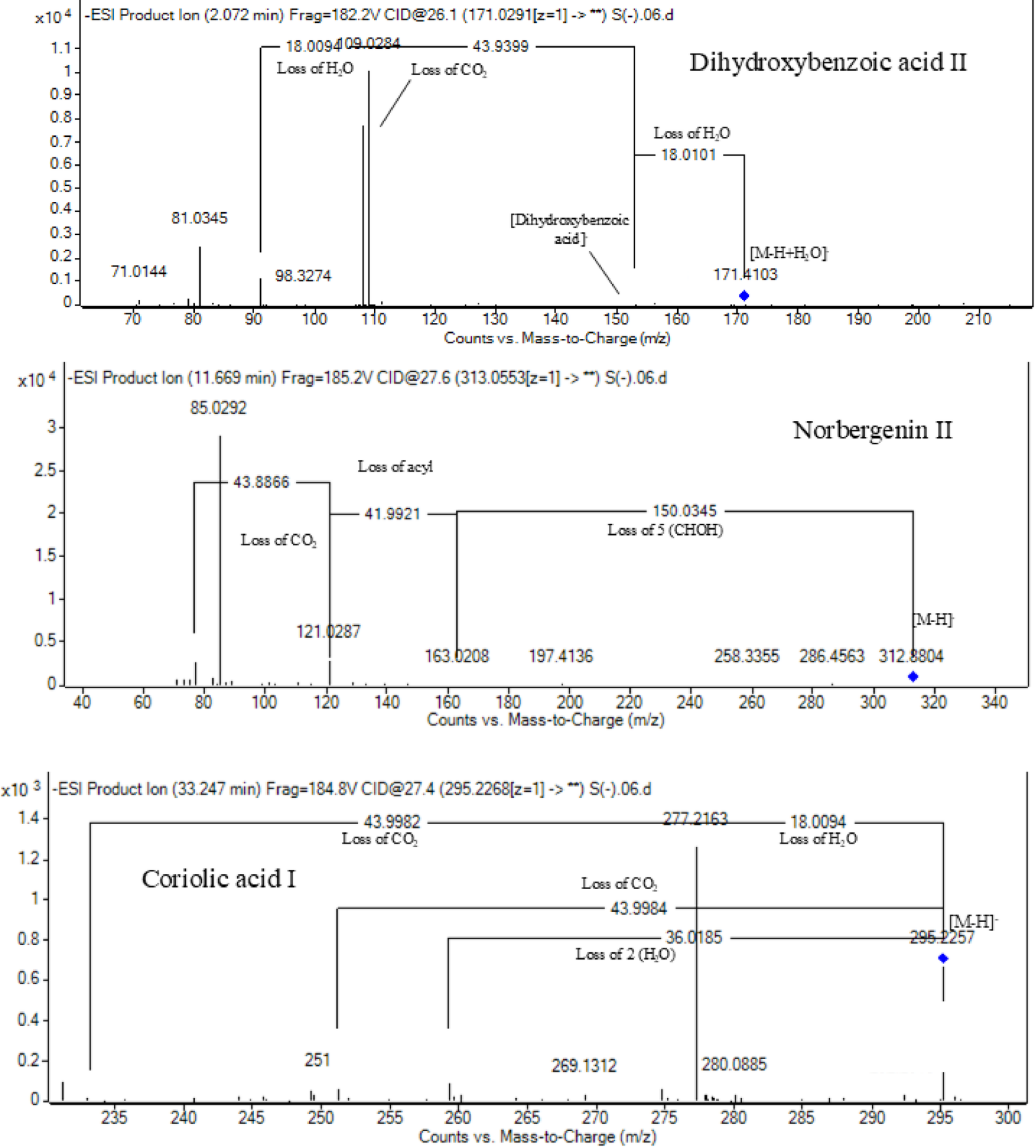
Pattern of fragmentation of (**a**) dihydroxybenzoic acid II, (**b**) norbergenin II, (**c**) coriolic acid I.

Peak 19 at RT at 1.893 min with the [M–H]^−^ at *m*/*z* 153.02 with predominant fragments at *m*/*z* 109.0291 [M–H–44] corresponding to decarboxylation the compound was identified as dihydroxybenzoic acid (Fig. 4)^57^ as the same pattern, Isomers at peak no 3,9,12 were identified as dihydroxybenzoic acid in the hydrated form. The absence of those peaks in the leaf extracts was clearly noticed. Dihydroxybenzoic acid was the first time to be identified in the genus *Swinglea*, but it was previously reported as 2,5-dihydroxybenzoic acid, 3,5-dihydroxybenzoic acid, and 3,4-dihydroxybenzoic acid (Protocatechuic acid) before in the family^58–60^.

Continuing with the class of hydroxybenzoic acids, gallic acid was identified in stem extract only at peak no 17 with RT 3.81 min and base peak [M–H]^−^ at *m*/*z* 169.0132 with diagnostic fragments at *m*/*z* 125.0230 [M–H–44]^−^ and 107.0149 [M–H–44-18]^−^ correspond to loss of CO_2_ and water ions^61,62^. To our knowledge, it’s the first time to be reported in the genus *Swinglea,* but reported before in the family^60^ (Table 3).

Peaks No 22 [M–H] at *m*/*z* 313.0549 showing fragment peak of 163.0235 at *m*/*z* with neutral loss of 150 Da (5*CHOH) followed by loss of acyl group [M–H-42]^−^ at 121.0288 *m*/*z* then decarboxylation with loss of 43.98 Da with fragment peak at 85.0293 *m/z* and the compound was tentatively identified as norbergenin (Fig. 4).

It is the first time to be reported in the genus *Swinglea,* but it was previously reported in the family Rutaceae^63^ (Table 3).

Peak no 24 with [M–H] at *m*/*z* 327.0707 showing fragments 267.0764, 209.0280, 167.0217, 121.0284 *m*/*z* corresponding to loss of acetate, then acyl group followed by decarboxylation; hence, the compound was tentatively identified as bergenin (Table 3).

#### II.II hydroxycinnamic acids

Peak 25 with RT 15.47 min and base peak [M–H]^−^ at *m*/*z* 355.1028 showing characteristic loss of hexose moiety 162.0551 Da giving fragment ion at *m*/*z* 193.0477 which diagnostic for ferulic acid followed by loss of methyl group then CO_2_ at *m*/*z* 178.0246 and *m*/*z* 134.0355 respectively so that it is identified as ferulic acid *O* hexose with another isomer at peak 28^64,65^. To our knowledge, it is the first time to be reported in the genus *Swinglea,* but noted before in the family^66^.

### II. Nonphenolic compounds

#### II.I. Organic acids

Loss of CO_2_ (M−H-44) with or without loss of one or more water ions is significant to the fragmentation pattern of organic acids. Accordingly, Peak no. 5,10,11,15, and 56 were identified as gluconic/galactonic acid.

Quinic acid, malic acid, Succinic acid, and azelaic acid, respectively to our knowledge, its first time have been reported in the genus, but they were previously reported in the family^67^ and with the same pattern, peaks no 2 and 7, which were only found in stem extract, were identified as Glucu/galacturonic acid I, II, with extra loss of 3*CHOH molecule after decarboxylation. Peak no 16 with base peak [M–H]^−^ at *m*/*z* 175.0239 was only detected in stem extract and was identified as Ascorbic acid, which is the first time to reported in the genus but previously reported in the family^68^ (Table 3).

#### II.II. Sialic acid derivatives

Peak no.14 which was detected in stem extract with base peak [M–H]^−^ at *m*/*z* 290.0872 with characteristic fragment ions at 200.0546 (M−H-90) and 170.0444 (M−H-120) followed by loss of acyl group 128.0342 (M−H-42) and was identified as 2-Deoxy-2,3-dehydro-*n*-acetyl-neuraminic acid^69,70^ and other isomers identified at RT 2.017 min. To our knowledge, it is the first time to be detected in the family Rutaceae.

#### II.III. Amino acids

Peak no 21 with base peak [M–H]^−^ at *m*/*z* 203.0817 with characteristic fragment at 142.0646 [M−H-61]^−^ which represents the loss of amino group 17 Da together with carboxylic group 44 Da which is a known pattern for amino acid fragmentation^71^ and the compound was identified as Tryptophan and was only detected in leaf extract and its first time to be recognized in a genus but reported previously in the family Rutaceae^72^ (Table 3).

#### II.IV. Sugars

Peaks no. 1 and 9 with [M–H]^−^ at *m*/z 179.06 were identified as monosaccharide I–II confirmed with characteristic neutral loss of 90 Da and 18 Da (neutral loss of water) and peak no 13 with [M–H]^−^ at *m*/*z* 149.04 was identified as pentose confirmed with characteristic neutral similar to the aforementioned compounds.

#### Fatty acids

Fifteen fatty acids derivatives were identified in leaves and stems of *Swinglea glutinosa* as long-chain fatty acids expressing dehydration and decarboxylation^73^; *viz.,* four isomers of hydroxylinolenic acid I–IV (*m/z* 293.21, C_18_H_30_O_3_) and hydroxystearic acid (*m/z* 299.26, C_18_H_36_O_3_) being characterized in the genus *Citrus*^55^. Moreover, two isomers of coriolic acid I–II (*m/z* 295.22, C_18_H_32_O_3_) (Fig. 4), and four isomers of ricinoleic acid I–IV (*m/z* 297.24, C_18_H_34_O_3_) were identified and previously mentioned in Rutaceae^55^. Also, four isomers of (9Z)-5,8,11-trihydroxyoctadec-9-enoic acid I–IV (*m/z* 329.23, C_18_H_34_O_5_) were noticed and mentioned in Rutaceae^55^ (Table 3).

### Intermolecular docking

The promising hit constituents of the stem extract (2-Deoxy-2,3-dehydro-*n*-acetyl-neuraminic acid, Ascorbic acid, Glucuronic acid, Protocatechuic acid, Galacturonic acid and Gallic acid) were subjected for further molecular modeling examination so as to predict and confirm their efficacy and mode of action against three important key isozymes (α-amylase enzyme [PDB id: 1b2y], α-glucosidase enzyme [PDB id: 3wy2] and acetylcholine esterase ACHE enzyme [PDB id: 6tt0]) that were involved in diabetes and Alzheimer diseases.

**Fig. 5:**
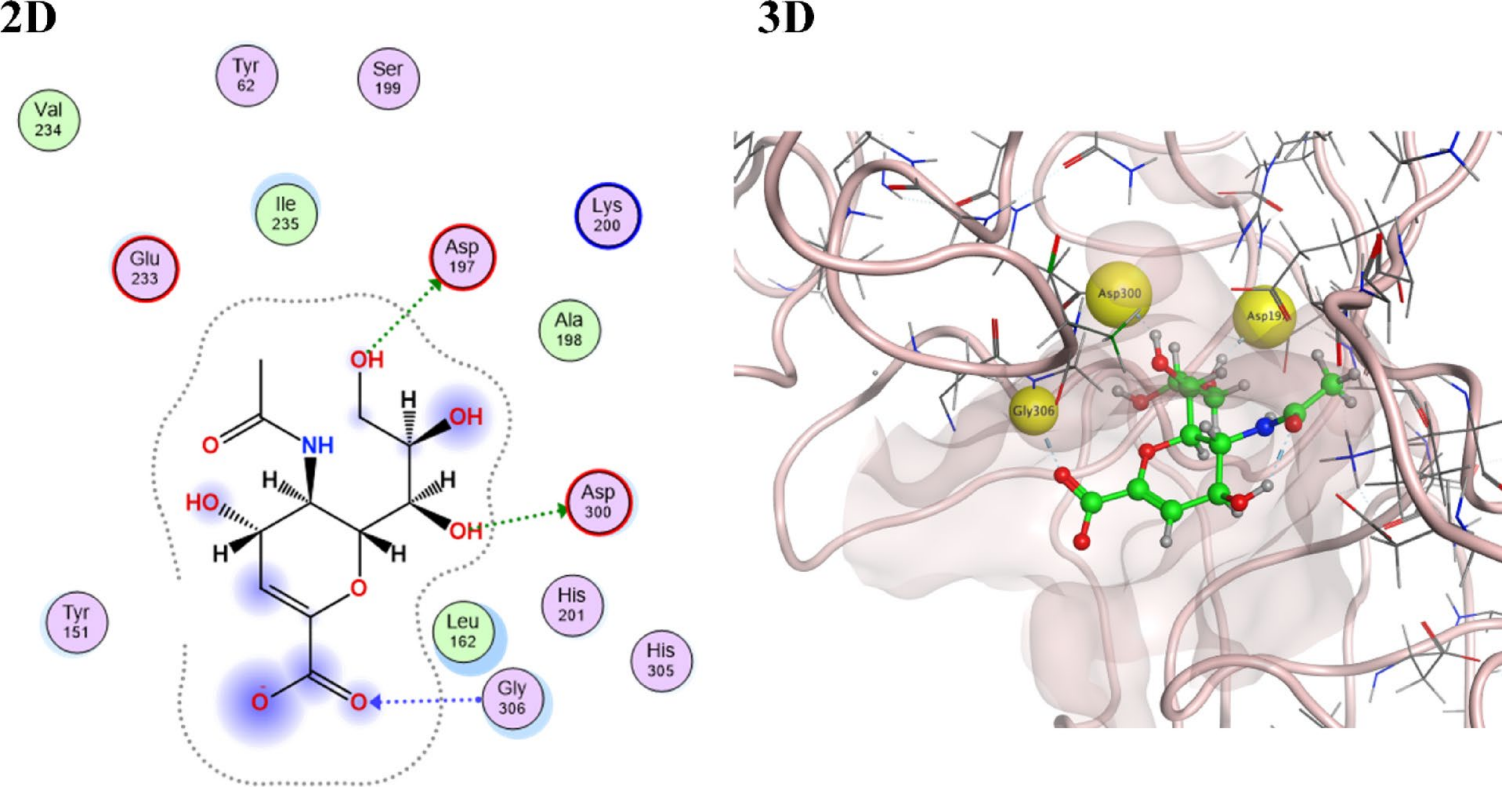
2-D and 3-D interaction docking poses for the highest score ranked stem extract component [2-Deoxy-2,3-dehydro-*N*-acetyl-neuraminic acid] against alpha-amylase enzyme (PDB id: 1b2y) using molecular operating environment software (MOE) 2019.0102 “*Molecular Operating Environment (MOE), 2024.0601 Chemical Computing Group ULC, 910-1010 Sherbrooke St. W., Montreal, QC H3A 2R7, 2025”*.

Concerning α-amylase enzyme, 2-Deoxy-2,3-dehydro-*N*-acetyl-neuraminic acid was the highest-ranked best-fitted compound with a scoring value of − 6.2531. It bound successfully to 3 main key amino acid residues of α-amylase enzymes Gly306, Asp197, and Asp300 via strong hydrogen bonding interactions (Table 4, Fig. 5). Compounds (Ascorbic acid and glucuronic acid) appeared as the second and third most highly ranked active constituents of the stem extract, showing binding scores equal to − 5.0406 and − 4.9790, respectively (Table 3). They performed a successful binding interaction, including strong hydrogen bonding and ionic electrostatic interactions with the key amino acids of the target enzyme Glu233, His201, and Lys200 (S1). Finally, compounds (Protocatechuic acid, Galacturonic acid, and Gallic acid) exhibited moderate binding score values of − 4.2616, − 4.1516, and − 4.1403, respectively, with a small number of strong hydrogen bonding and ionic interactions with respect to key residues.

**Table 4:** Docking scores and featured interaction of the target components of the stem extract of *Swinglea glutinosa* against alpha-amylase enzyme (PDB id: 1b2y).

Compound	S-score (kcal/mol)	Involved receptor residues	Type of bond interaction
2-Deoxy-2,3-dehydro-*n*-acetyl-neuraminic acid	− 6.2531	Gly306	H-bonding
		Asp197	H-bonding
		Asp300	H-bonding
Ascorbic acid	− 5.0406	Glu233	H-bonding
		Ile235	H-bonding
		His201	H-bonding
Glucuronic acid	− 4.9790	Glu233	H-bonding
		Ile235	H-bonding
		His201	H-bonding
		Lys200	H-bonding
		Lys200	Ionic interaction
Protocatechuic acid	− 4.2616	Glu233	H-bonding
		Asp300	H-bonding
		His101	Ionic interaction
Galacturonic acid	− 4.1516	Glu233	H-bonding
		His101	Ionic interaction
Gallic acid	− 4.1403	Asp300	H-bonding
		His299	H-bonding
		His101	Ionic interaction

**Fig. 6:**
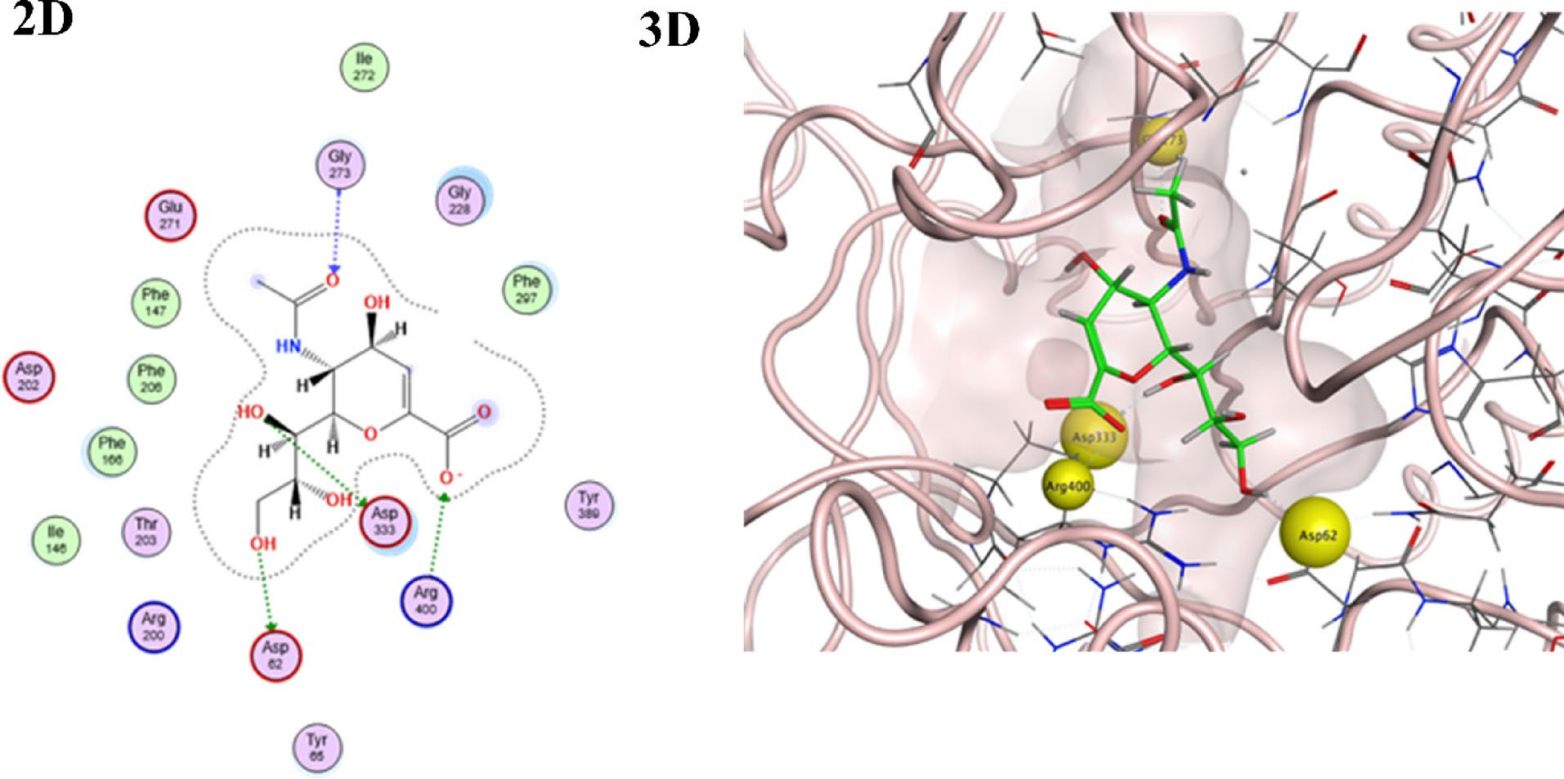
2-D and 3-D interaction docking poses for the highest score ranked stem extract component [2-Deoxy-2,3-dehydro-*N*-acetyl-neuraminic acid] against α-glucosidase enzyme (PDB id: 3wy2) using molecular operating environment software (MOE) 2019.0102 “*Molecular Operating Environment (MOE), 2024.0601 Chemical Computing Group ULC, 910-1010 Sherbrooke St. W., Montreal, QC H3A 2R7, 2025”*.

Studying the protein–ligand complexes of this series of the stem active constituents against α-glucosidase enzyme represented 2-Deoxy-2,3-dehydro-*n*-acetyl-neuraminic acid as the most effective compound due to the elevated enzyme-binding score − 6.0038 kcal/mol in addition to the formation of strong hydrogen bonds with the key residues of α-glucosidase enzyme (Asp333, Asp62, and Arg400) (Table 5, Fig. 6). Moreover, compounds (Ascorbic acid and Glucuronic acid) were the second and third most active constituents of the stem extract, with binding scores of − 5.6515 and − 5.4445, respectively (Table 5). They successfully bound to the target enzyme’s critical amino acids Glu271, Asp202, Asp62, Arg400, Phe166, and Asp333 via strong hydrogen bonding and ionic interactions (S2). Finally, (Galacturonic acid, Gallic acid, and Protocatechuic acid) had modest binding score values of − 5.0975, − 4.2409, and − 4.1665, respectively (Table 4).

**Table 5:** Docking scores and featured interaction of the target components of the stem extract of *Swinglea glutinosa* against α-glucosidase enzyme (PDB id: 3wy2).

Compound	S-score (kcal/mol)	Involved receptor residues	Type of bond interaction
2-Deoxy-2,3-dehydro-*N*-acetyl-neuraminic acid	− 6.0038	Gly273	H-bonding
		Asp333	H-bonding
		Asp62	H-bonding
		Arg400	H-bonding
Ascorbic acid	− 5.6515	Glu271	H-bonding
		Asp202	H-bonding
		Asp62	H-bonding
		Arg400	H-bonding
		Phe166	Hydrophobic interaction
Glucuronic acid	− 5.4445	Asp202	H-bonding
		Asp62	H-bonding
		Asp333	2-H-bonding
		Arg400	Ionic interaction
Galacturonic acid	− 5.0975	Asp202	2-H-bonding
		Asp62	H-bonding
		Arg400	H-bonding
		Asp333	H-bonding
		Arg400	Ionic interaction
Gallic acid	− 4.2409	Glu271	H-bonding
		Arg200	H-bonding
Protocatechuic acid	− 4.1665	Glu271	H-bonding

**Fig. 7:**
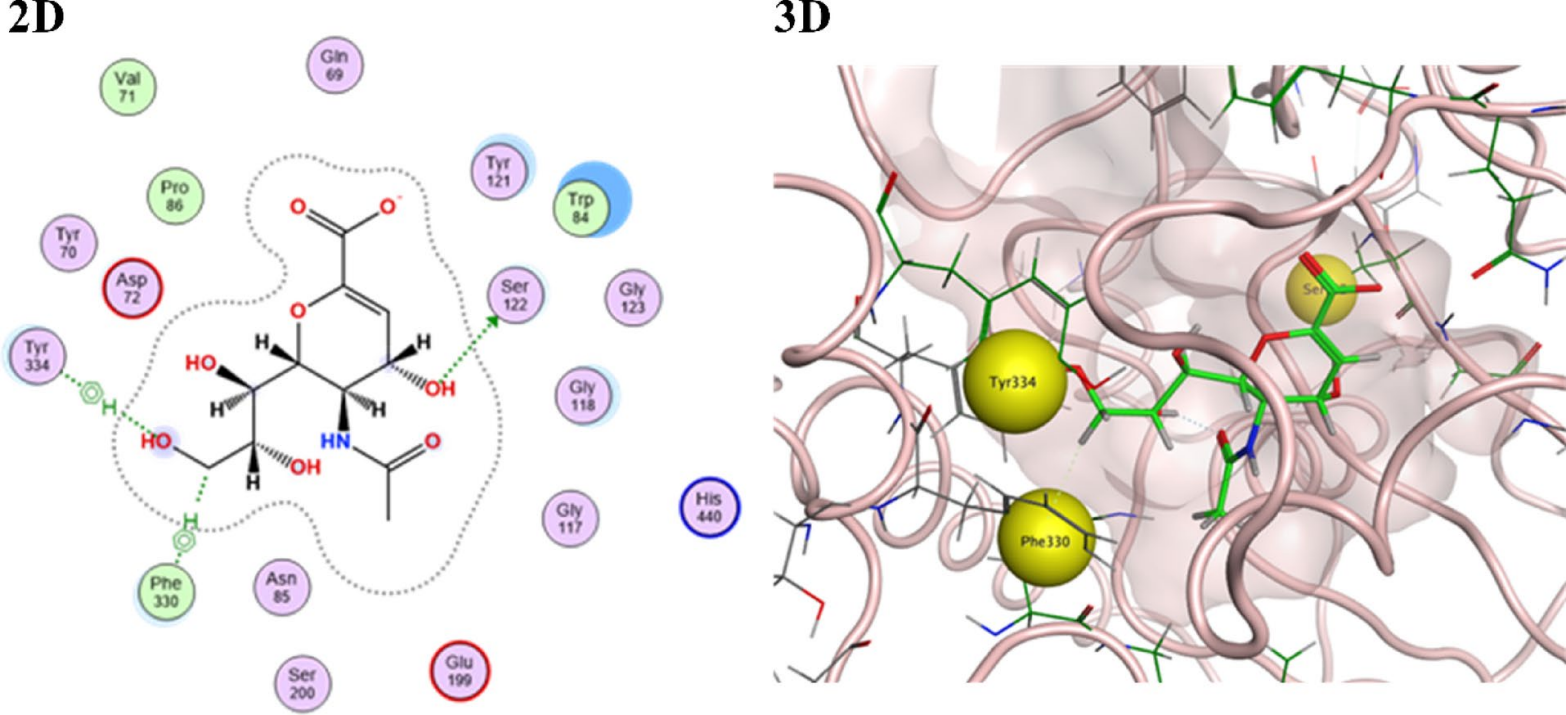
2-D and 3-D interaction docking poses for the highest score ranked stem extract component [2-Deoxy-2,3-dehydro-*N*-acetyl-neuraminic acid] against acetyl choline esterase ACHE enzyme (PDB id: 6tt0) using molecular operating environment software (MOE) 2019.0102 “*Molecular Operating Environment (MOE), 2024.0601 Chemical Computing Group ULC, 910-1010 Sherbrooke St. W., Montreal, QC H3A 2R7, 2025”*.

Regarding Alzheimer acetyl choline esterase ACHE enzyme, 2-Deoxy-2,3-dehydro-*N*-acetyl-neuraminic acid preside the whole series. It revealed the best binding score (− 5.9556 kcal/mol). Moreover, it fulfilled the active site of the enzyme through various attachments including hydrogen bonds and hydrophobic interactions to the key amino acid residues of the enzyme (Table 6, Fig. 7). While the other constituents of the stem extract were ranked according to their binding scores to the enzyme as Ascorbic acid (− 4.6331), Glucuronic acid (− 4.6196), Gallic acid (− 4.5612), Galacturonic acid (− 4.5436) and Protocatechuic acid (− 4.4075) (Table 5, Fig. 7). Their moderate binding scores predict their moderate activity.

**Table 6:** Docking scores and featured interaction of the target components of the stem extract of *Swinglea glutinosa* against acetyl choline esterase ACHE enzyme (PDB id: 6tt0).

Compound	S-score (kcal/mol)	Involved receptor residues	Type of bond interaction
2-Deoxy-2,3-dehydro-*N*-acetyl-neuraminic acid	− 5.9556	Ser122	H-bonding
		Phe330	H-bonding
		Tyr334	Hydrophobic interaction
Ascorbic acid	− 4.6331	Glu199	H-bonding
Glucuronic acid	− 4.6196	Glu199	H-bonding
		His440	Ionic interaction
Gallic acid	− 4.5612	Glu199	H-bonding
		His440	Ionic interaction
Galacturonic acid	− 4.5436	His440	Ionic interaction
Protocatechuic acid	− 4.4075	Glu199	2-H-bonding
		His440	Ionic interaction

#### Molecular dynamic simulation

**Fig. 8:**
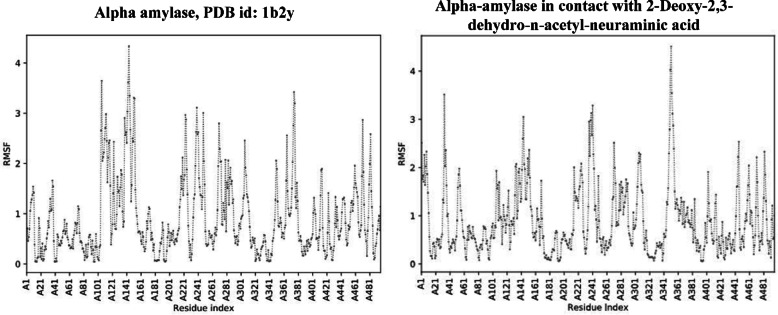
RMSF of Enzyme (alpha amylase, PDB id: 1b2y) RMSF of Enzyme-Ligand complex (alpha-amylase in contact with 2-Deoxy-2,3-dehydro-*n*-acetyl-neuraminic acid).

The docking study revealed that compound 2-Deoxy-2,3-dehydro-*N*-acetyl-neuraminic acid was clearly the most potent and best-ranked active constituent in the stem extract, exhibiting the highest binding scores and the best-fitting poses. Consequently, a molecular dynamic simulation was performed to validate and prove the docking results. Studying the root mean square fluctuation (RMSF) charts of the enzyme (Fig. 8) and the enzyme-ligand complex (Fig. 8) showed great flexibility of the enzyme amino acid residues that tend to decrease their fluctuating movements after binding to the active constituent 2-Deoxy-2,3-dehydro-*N*-acetyl-neuraminic acid indicating the stabilization of the enzyme-ligand complex and proving the inhibitory effect of 2-Deoxy-2,3-dehydro-*N*-acetyl-neuraminic acid.

**Fig. 9:**
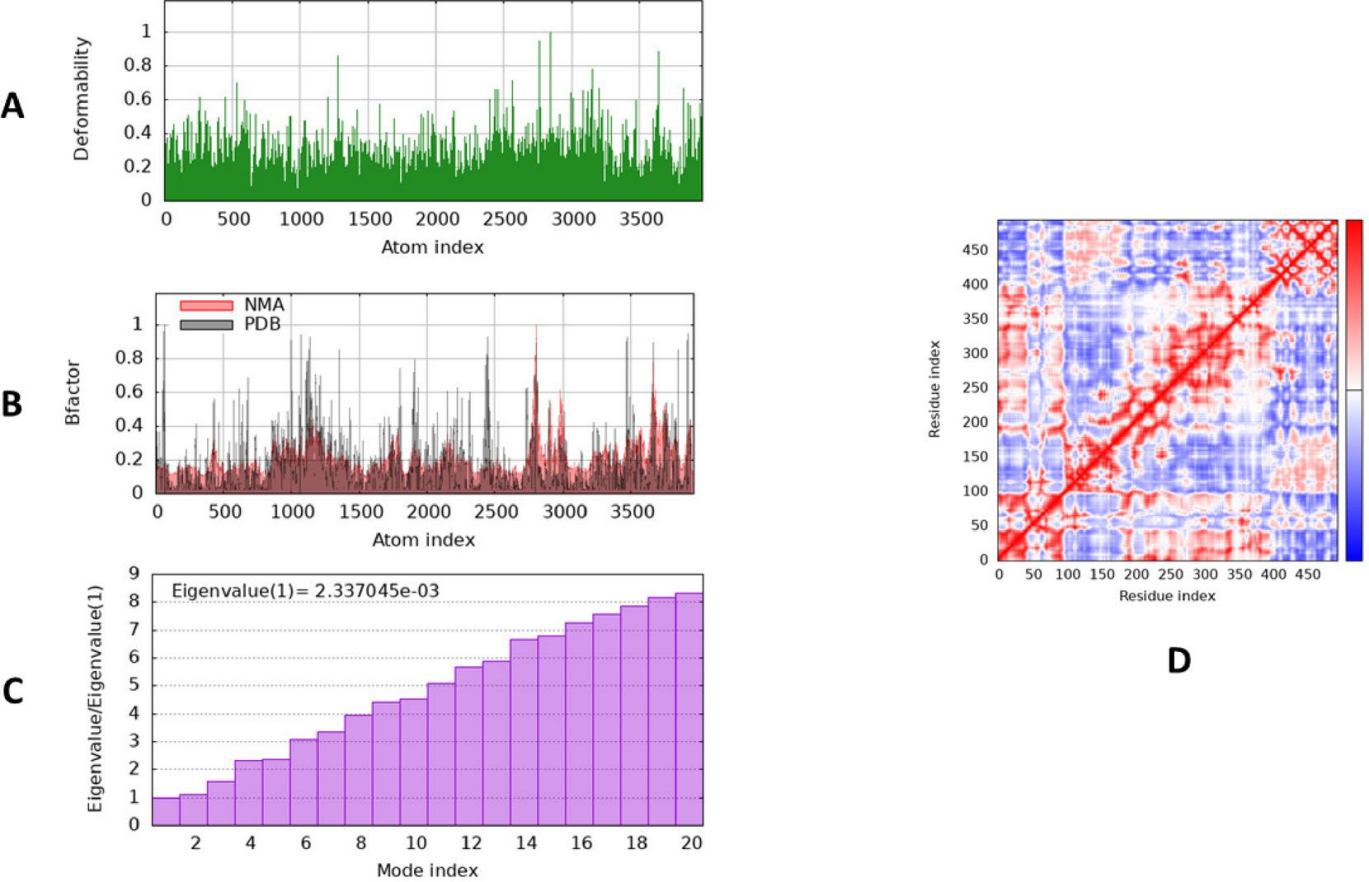
Molecular dynamics simulation of Enzyme-Ligand complex (alpha-amylase in contact with 2-Deoxy-2,3-dehydro-*n-*acetyl-neuraminic acid) by iMODS server. (**A**) Deformability, (**B**) B-factor values, (**C**) eigenvalue and (**D**) covariance model.

To evaluate the physical movement and stability of our ligand–protein complex, we performed MD simulation using IMODS. (NMA) normal mode analysis was conducted to investigate the stability of our docked complexes. NMA of our docked complex is illustrated in Fig. 9. The main-chain deformability graph shown in Fig. 9A illustrating that higher peaks represent the regions of the protein deformability which indicate the flexibility and relocation of group of amino acid resides so as to improve their binding to the target compound (Fig. 9A). The experimental B-factor in Fig. 9B determines the exact position of the atoms. The atomic positions always form irregularities in the crystal form. The higher the B-factor value, the higher the mobility and flexibility of a crystal^3^. The B-factor graph indicate the higher degree of protein residues fluctuations along the atom index ranges of 1000 and 3500. It was clear that the ligand-enzyme complex for compound (2-Deoxy-2,3-dehydro-*n*-acetyl-neuraminic acid) had quite low eigenvalue of 2.337045e−03, which reveal better ability of deformability and a high degree of protein flexibility (Fig. 9C). The covariance map (Fig. 9D) between the ligand and enzyme residues is indicating their high correlations (correlated motion indicated by red color; uncorrelated motion indicated by white color; anti-correlated motion indicated by blue color).

Generally, docking results revealed that all the selected compounds of the stem extract exhibited high fitting scores and various binding solid interactions with the key amino acid residues of the selected target enzymes. However, the greatest findings were obtained against the alpha-amylase enzyme (PDB id: 1b2y), which was matched with the experimental biological assay investigation. Interestingly, 2-Deoxy-2,3-dehydro-*N*-acetyl-neuraminic acid, Ascorbic acid, and Glucuronic acid preside appeared as the highly ranked active constituents against all target enzymes, respectively. Finally, it was obvious that compound 2-Deoxy-2,3-dehydro-*N*-acetyl-neuraminic acid was the best highly ranked potent compound of the stem extract showing the best fitting poses and the highest binding scores. This evidence was supported by the results of the molecular dynamic simulation study that succeeded in proving the effect of 2-Deoxy-2,3-dehydro-*N*-acetyl-neuraminic acid against the α-amylase enzyme via decreasing the fluctuations of the enzyme’s amino acid residues due to stabilization of enzyme-ligand complex.

## Conclusion

For additional investigations on the stem extract’s higher ability to inhibit the enzymes cholinesterase, α-glucosidase, and amylase compared to leaves. Intermolecular docking was used to test six phytoconstituents compounds that were found only in stems: 2-Deoxy-2,3-dehydro-*N*-acetyl-neuraminic acid (DANA), Ascorbic acid, Glucuronic acid, Protocatechuic acid, Galacturonic acid, and Gallic acid. The results of the test showed that all of the compounds had highly fitting scores against all of the targeted enzymes, with DANA, Ascorbic acid, and Glucuronic acid having the highest rank, respectively. A molecular dynamic simulation study for DANA proceeded and succeeded in proving the effect of DANA on the alpha-amylase enzyme by decreasing the fluctuations of the enzyme’s amino acid residues due to the stabilization of the enzyme-ligand complex. Antidiabetic activity and managing Alzheimer’s dementia were correlated to the aforementioned tested compounds.

## Electronic supplementary material

Below is the link to the electronic supplementary material.

Supplementary Material 1

## Data Availability

The authors declare that the data supporting this study’s findings are available within the paper. Data is provided within the manuscript and the supplementary materials.

## References

[1] Mauricio, D., Alonso, N. & Gratacòs, M. Chronic diabetes complications: The need to move beyond classical concepts. *Trends Endocrinol. Metab.* **31**, 287–295 (2020). 32033865 10.1016/j.tem.2020.01.007

[2] Park, H. C. et al. Diabetic retinopathy is a prognostic factor for progression of chronic kidney disease in the patients with type 2 diabetes mellitus. *PLoS One* **14**, e0220506 (2019). 31356653 10.1371/journal.pone.0220506 PMC6663021

[3] Abouzed, T. K. et al. Red onion scales ameliorated streptozotocin-induced diabetes and diabetic nephropathy in Wistar rats in relation to their metabolite fingerprint. *Diabetes Res. Clin. Pract.* **140**, 253–264. 10.1016/j.diabres.2018.03.042 (2018). 29626589 10.1016/j.diabres.2018.03.042

[4] Gong, L. et al. Inhibitors of α-amylase and α-glucosidase: Potential linkage for whole cereal foods on prevention of hyperglycemia. *Food Sci. Nutr.* **8**, 6320–6337 (2020). 33312519 10.1002/fsn3.1987 PMC7723208

[5] Amin, E. et al. The glycemic control potential of some Amaranthaceae plants, with particular reference to in vivo antidiabetic potential of *Agathophora**alopecuroides*. *Molecules* **27**, 973 (2022). 35164238 10.3390/molecules27030973 PMC8839903

[6] Markesbery, W. R. Oxidative stress hypothesis in Alzheimer’s disease. *Free Radic. Biol. Med.* **23**, 134–147 (1997). 9165306 10.1016/s0891-5849(96)00629-6

[7] Talesa, V. N. Acetylcholinesterase in Alzheimer’s disease. *Mech. Ageing Dev.* **122**, 1961–1969 (2001). 11589914 10.1016/s0047-6374(01)00309-8

[8] Shrivastava, S. K. et al. Design and development of novel p-aminobenzoic acid derivatives as potential cholinesterase inhibitors for the treatment of Alzheimer’s disease. *Bioorg. Chem.* **82**, 211–223 (2019). 30326403 10.1016/j.bioorg.2018.10.009

[9] Mani, V. et al. Sukkari dates seed improves type-2 diabetes mellitus-induced memory impairment by reducing blood glucose levels and enhancing brain cholinergic transmission: In vivo and molecular modeling studies. *Saudi Pharm. J.* **30**, 750–763. 10.1016/j.jsps.2022.03.016 (2022). 35812141 10.1016/j.jsps.2022.03.016 PMC9257867

[10] Abdalla, M. M. I. Insulin resistance as the molecular link between diabetes and Alzheimer’s disease. *World J. Diabetes* **15**, 1430–1447. 10.4239/wjd.v15.i7.1430 (2024). 39099819 10.4239/wjd.v15.i7.1430 PMC11292327

[11] Mohammed, H. A. et al. Essential oils pharmacological activity: Chemical markers, biogenesis, plant sources, and commercial products. *Process Biochem.* **144**, 112–132. 10.1016/j.procbio.2024.05.021 (2024).

[12] Sima, A. A. & Li, Z. G. Diabetes and Alzheimer’s disease—Is there a connection?. *Rev. Diabet. Stud.* **3**, 161–168. 10.1900/rds.2006.3.161 (2006). 17487340 10.1900/RDS.2006.3.161 PMC1828287

[13] Gratuze, M., Julien, J., Petry, F. R., Morin, F. & Planel, E. Insulin deprivation induces PP2A inhibition and tau hyperphosphorylation in hTau mice, a model of Alzheimer’s disease-like tau pathology. *Sci. Rep.* **7**, 46359. 10.1038/srep46359 (2017). 28402338 10.1038/srep46359 PMC5389355

[14] Ayaz, M. et al. Antioxidant, enzyme inhibitory, and molecular docking approaches to the antidiabetic potentials of bioactive compounds from *Persicaria**hydropiper* L. *Evid.-Based Complement. Altern. Med.* **2022**, 6705810. 10.1155/2022/6705810 (2022). 10.1155/2022/6705810 PMC9023165 35463090

[15] Elshibani, F., Alamami, A., Khan, R., Sulaiman, G. M. & Mohammed, H. A. *Haplophyllum**tuberculatum* (Forssk.) A. Juss essential oils: Seasonal contents variation, bioactivity of the traditionally-favored, high-yield and frequent-use summer season oil. *J. Oleo Sci.* **73**, 263–273. 10.5650/jos.ess23055 (2024). 38233115 10.5650/jos.ess23055

[16] Huang, L., Feng, Z.-L., Wang, Y.-T. & Lin, L.-G. Anticancer carbazole alkaloids and coumarins from Clausena plants: A review. *Chin. J. Nat. Med.* **15**, 881–888. 10.1016/S1875-5364(18)30003-7 (2017). 29329644 10.1016/S1875-5364(18)30003-7

[17] Fatema-Tuz-Zohora, H. C. & Ahsan, M. Chemical constituents, cytotoxic activities and traditional uses of Micromelum minutum (Rutaceae): A review. *Pharm. Pharmacol. Int. J.* **7**, 229–236 (2019).

[18] Tamokou, J. D. D., Mbaveng, A. T. & Kuete, V. In *Medicinal Spices and Vegetables from Africa* (ed Kuete, V.) 207–237 (Academic Press, 2017).

[19] León, M. G., Becerra, C. H., Freitas-Astúa, J., Salaroli, R. & Elliot, K. Natural infection of *Swinglea**glutinosa* by the *Citrus**leprosis* virus cytoplasmic type (CiLV-C) in Colombia. *Plant Dis.* **92**, 1364–1364. 10.1094/PDIS-92-9-1364C (2008). 30769432 10.1094/PDIS-92-9-1364C

[20] Weniger, B. et al. Bioactive acridone alkaloids from *Swinglea**glutinosa*. *J. Nat. Prod.* **64**, 1221–1223 (2001). 11575960 10.1021/np0005762

[21] Stashenko, E., Martínez, J. R., Medina, J. D. & Durán, D. C. Analysis of essential oils isolated by steam distillation from *Swinglea**glutinosa* fruits and leaves. *J. Essent. Oil Res.* **27**, 276–282 (2015).

[22] Braga, P. et al. In vitro cytotoxicity activity on several cancer cell lines of acridone alkaloids and N-phenylethyl-benzamide derivatives from *Swinglea**glutinosa* (Bl.) Merr. *Nat. Prod. Res.* **21**, 47–55 (2007). 17365689 10.1080/14786410600907002

[23] Žilić, S., Serpen, A., Akıllıoğlu, G. L., Gökmen, V. & Vančetović, J. Phenolic compounds, carotenoids, anthocyanins, and antioxidant capacity of colored maize (*Zea**mays* L.) kernels. *J. Agric. Food Chem.* **60**, 1224–1231 (2012). 10.1021/jf204367z 22248075

[24] Hassanin, S. O. et al. Combining in vitro, in vivo, and network pharmacology assays to identify targets and molecular mechanisms of Spirulina-derived biomolecules against breast cancer. *Mar. Drugs* **22**, 328 (2024). 39057437 10.3390/md22070328 PMC11278317

[25] Sweilam, S. H., Abd El Hafeez, M. S., Mansour, M. A. & Mekky, R. H. Unravelling the phytochemical composition and antioxidant potential of different parts of *Rumex**vesicarius* L.: A RP-HPLC-MS-MS/MS, chemometrics, and molecular docking-based comparative study. *Plants* **13**, 1815 (2024). 38999655 10.3390/plants13131815 PMC11244572

[26] Abd El Hafeez, M. S., El Gindi, O., Hetta, M. H., Aly, H. F. & Ahmed, S. A. Quality control, anti-hyperglycemic, and anti-inflammatory assessment of *Colvillea**racemosa* leaves using in vitro, in vivo investigations and its correlation with the phytoconstituents Identified via LC-QTOF-MS and MS/MS. *Plants* **11**, 830 (2022). 35336712 10.3390/plants11060830 PMC8948708

[27] Ingkaninan, K., Temkitthawon, P., Chuenchom, K., Yuyaem, T. & Thongnoi, W. Screening for acetylcholinesterase inhibitory activity in plants used in Thai traditional rejuvenating and neurotonic remedies. *J. Ethnopharmacol.* **89**, 261–264 (2003). 14611889 10.1016/j.jep.2003.08.008

[28] Saeed, N. M. et al. Exploring the anti-osteoporosis potential of *Petroselinum**crispum* (Mill.) Fuss extract employing experimentally ovariectomized rat model and network pharmacology approach. *Fitoterapia* **175**, 105971. 10.1016/j.fitote.2024.105971 (2024). 38663562 10.1016/j.fitote.2024.105971

[29] Tej, A. et al. *Eucalyptus torquata* seeds: Investigation of phytochemicals profile via LC-MS and its potential cardiopreventive capacity in rats. *Food Biosci.* **59**, 103666. 10.1016/j.fbio.2024.103666 (2024).

[30] Mekky, R. H., Abdel-Sattar, E., Segura-Carretero, A. & Contreras, M. D. M. Phenolic compounds from sesame cake and antioxidant activity: A new insight for agri-food residues’ significance for sustainable development. *Foods* **8**, 432 (2019). 31546743 10.3390/foods8100432 PMC6835672

[31] Mekky, R. H. et al. Profiling of phenolic and other compounds from Egyptian cultivars of chickpea (*Cicer**arietinum* L.) and antioxidant activity: A comparative study. *RSC Adv.* **5**, 17751–17767 (2015).

[32] Mekky, R. H. et al. Comparative metabolite profiling and antioxidant potentials of seeds and sprouts of three Egyptian cultivars of *Vicia faba* L. *Food Res. Int.* **136**, 109537. 10.1016/j.foodres.2020.109537 (2020). 32846596 10.1016/j.foodres.2020.109537

[33] Fawzy, M. G. & Said, M. A. Valuation of environmental influence of recently invented high-performance liquid chromatographic method for hypoglycemic mixtures of gliflozins and metformin in the presence of melamine impurities: Application of molecular modeling simulation approach. *J. Sep. Sci.* **46**, 2300267 (2023). 10.1002/jssc.202300267 37485588

[34] Said, M. A., Albohy, A., Abdelrahman, M. A. & Ibarhim, H. S. Remdesivir analog as SARS-CoV-2 polymerase inhibitor: Virtual screening of a database generated by scaffold replacement. *RSC Adv.* **12**, 22448–22457 (2022). 36105996 10.1039/d2ra00486k PMC9366421

[35] Hassan, A. S., Morsy, N. M., Aboulthana, W. M. & Ragab, A. In vitro enzymatic evaluation of some pyrazolo [1, 5-a] pyrimidine derivatives: Design, synthesis, antioxidant, anti-diabetic, anti-Alzheimer, and anti-arthritic activities with molecular modeling simulation. *Drug Dev. Res.* **84**, 3–24 (2023). 36380556 10.1002/ddr.22008

[36] Kuriata, A. et al. CABS-flex 2.0: A web server for fast simulations of flexibility of protein structures. *Nucleic Acids Res.* **46**, W338–W343 (2018). 29762700 10.1093/nar/gky356 PMC6031000

[37] Abirami, A., Nagarani, G. & Siddhuraju, P. In vitro antioxidant, anti-diabetic, cholinesterase and tyrosinase inhibitory potential of fresh juice from *Citrus**hystrix* and *C*. *maxima* fruits. *Food Sci. Hum. Wellness* **3**, 16–25. 10.1016/j.fshw.2014.02.001 (2014).

[38] Chaiyana, W. & Okonogi, S. Inhibition of cholinesterase by essential oil from food plant. *Phytomedicine* **19**, 836–839. 10.1016/j.phymed.2012.03.010 (2012). 22510493 10.1016/j.phymed.2012.03.010

[39] Feitosa, C. et al. Antioxidant and anticholinergic properties of *Citrus**sinensis* (L.) Osbeck (Rutaceae) essential oil in mice hippocampus (2020).

[40] Wszelaki, N., Kuciun, A. & Kiss, A. Screening of traditional European herbal medicines for acetylcholinesterase and butyrylcholinesterase inhibitory activity. *Acta Pharm.* **60**, 119 (2010). 20228046 10.2478/v10007-010-0006-y

[41] Dwivedi, P. S., Rasal, V., Chavan, R. S., Khanal, P. & Gaonkar, V. P. Feronia *elephantum* reverses insulin resistance in fructose-induced hyper-insulinemic rats; An in-silico, in-vitro, and in-vivo approach. *J. Ethnopharmacol.* **316**, 116686 (2023). 37279812 10.1016/j.jep.2023.116686

[42] Trinh, D. H. et al. Coumarins and acridone alkaloids with α-glucosidase inhibitory and antioxidant activity from the roots of *Paramignya**trimera*. *Phytochem. Lett.* **35**, 94–98. 10.1016/j.phytol.2019.10.010 (2020).

[43] Mekky, R. H., Abdel-Sattar, E., Segura-Carretero, A. & Contreras, M. D. M. Metabolic profiling of the oil of sesame of the Egyptian cultivar ‘Giza 32’ employing LC-MS and tandem MS-based untargeted method. *Foods* **10**, 298 (2021). 33540686 10.3390/foods10020298 PMC7913063

[44] Mekky, R. H., Abdel-Sattar, E., Segura-Carretero, A. & Contreras, M. D. M. A comparative study on the metabolites profiling of linseed cakes from Egyptian cultivars and antioxidant activity applying mass spectrometry-based analysis and chemometrics. *Food Chem.* **395**, 133524. 10.1016/j.foodchem.2022.133524 (2022). 35878508 10.1016/j.foodchem.2022.133524

[45] Cao, J., Yin, C., Qin, Y., Cheng, Z. & Chen, D. Approach to the study of flavone di-C-glycosides by high performance liquid chromatography-tandem ion trap mass spectrometry and its application to characterization of flavonoid composition in *Viola**yedoensis*. *J. Mass Spectrom.* **49**, 1010–1024. 10.1002/jms.3413 (2014). 25303391 10.1002/jms.3413

[46] Afifi, S. M., Kabbash, E. M., Berger, R. G., Krings, U. & Esatbeyoglu, T. Comparative untargeted metabolic profiling of different parts of *Citrus**sinensis* fruits via liquid chromatography–mass spectrometry coupled with multivariate data analyses to unravel authenticity. *Foods* **12**, 579 (2023). 36766108 10.3390/foods12030579 PMC9914239

[47] Hirawan, R. & Beta, T. C-Glycosylflavone and lignan diglucoside contents of commercial, regular, and whole-wheat spaghetti. *Cereal Chem.* **88**, 338–343. 10.1094/CCHEM-01-10-0010 (2011).

[48] Mekky, R. H. et al. Metabolic profiling and antioxidant activity of fenugreek seeds cultivars ‘Giza 2’ and ‘Giza 30’ compared to other geographically-related seeds. *Food Chem. X* **24**, 101819. 10.1016/j.fochx.2024.101819 (2024). 39328377 10.1016/j.fochx.2024.101819 PMC11426063

[49] Candela, L., Formato, M., Crescente, G., Piccolella, S. & Pacifico, S. Coumaroyl flavonol glycosides and more in marketed green teas: An intrinsic value beyond much-lauded catechins. *Molecules* **25**, 1765 (2020). 32290396 10.3390/molecules25081765 PMC7221963

[50] Abu-Reidah, I. M. et al. HPLC–ESI-Q-TOF-MS for a comprehensive characterization of bioactive phenolic compounds in cucumber whole fruit extract. *Food Res. Int.* **46**, 108–117 (2012).

[51] Haggag, E., Mahmoud, I., Abou-Moustafa, E. & Mabry, T. Flavonoids from the leaves of *Citrus**aurantium* (sour orange) and *Citrus**sinensis* (sweet orange). *Asian J. Chem.* **11**, 707–714 (1999).

[52] Farias, K. D. S. et al. In depth investigation of the metabolism of *Nectandra**megapotamica* chemotypes. *PLoS One* **13**, e0201996 (2018). 30080887 10.1371/journal.pone.0201996 PMC6078319

[53] Chopin, J., Dellamonica, G., Markham, K., Nair, A. R. & Gunasegaran, R. 2″-p-coumaroylvitexin 7-glucoside from *Mollugo**oppositifolia*. *Phytochemistry* **23**, 2106–2108 (1984).

[54] Peña-Vázquez, G. I. et al. In vitro simulated gastrointestinal digestion impacts bioaccessibility and bioactivity of Sweet orange (*Citrus**sinensis*) phenolic compounds. *J. Funct. Foods* **88**, 104891. 10.1016/j.jff.2021.104891 (2022).

[55] Reaxys. http://www.reaxys.com.

[56] Chen, J. et al. Mechanisms of Lian-Gui-Ning-Xin-Tang in the treatment of arrhythmia: Integrated pharmacology and in vivo pharmacological assessment. *Phytomedicine* **99**, 153989 (2022). 35272242 10.1016/j.phymed.2022.153989

[57] Quifer-Rada, P. et al. A comprehensive characterisation of beer polyphenols by high resolution mass spectrometry (LC–ESI-LTQ-Orbitrap-MS). *Food Chem.* **169**, 336–343. 10.1016/j.foodchem.2014.07.154 (2015). 25236235 10.1016/j.foodchem.2014.07.154

[58] El-Sayed, M. A., Al-Gendy, A. A., Hamdan, D. I. & El-Shazly, A. M. Phytoconstituents, LC-ESI-MS profile, antioxidant and antimicrobial activities of Citrus x limon L. Burm. F. cultivar variegated pink lemon. *J. Pharm. Sci. Res.* **9**, 375 (2017).

[59] Wang, E. et al. Separation and enrichment of phenolics improved the antibiofilm and antibacterial activity of the fractions from *Citrus**medica* L. var. *sarcodactylis* in vitro and in tofu. *Food Chem.* **294**, 533–538 (2019). 31126496 10.1016/j.foodchem.2019.05.038

[60] Zahoor, S., Anwar, F., Mehmood, T., Sultana, B. & Qadir, R. Variation in antioxidant attributes and individual phenolics of citrus fruit peels in relation to different species and extraction solvents. *J. Chil. Chem. Soc.* **61**, 2884–2889 (2016).

[61] Heras, R.M.-L., Quifer-Rada, P., Andrés, A. & Lamuela-Raventós, R. Polyphenolic profile of persimmon leaves by high resolution mass spectrometry (LC-ESI-LTQ-Orbitrap-MS). *J. Funct. Foods* **23**, 370–377. 10.1016/j.jff.2016.02.048 (2016).

[62] Hossain, M. B., Rai, D. K., Brunton, N. P., Martin-Diana, A. B. & Barry-Ryan, C. Characterization of phenolic composition in Lamiaceae spices by LC-ESI-MS/MS. *J. Agric. Food Chem.* **58**, 10576–10581 (2010). 20825192 10.1021/jf102042g

[63] Razola-Díaz, M. D. C. et al. Optimization of ultrasound-assisted extraction via sonotrode of phenolic compounds from orange by-products. *Foods* **10**, 1120 (2021). 34070065 10.3390/foods10051120 PMC8158112

[64] Kajdžanoska, M., Gjamovski, V. & Stefova, M. HPLC-DAD-ESI-MSn identification of phenolic compounds in cultivated strawberries from Macedonia. *Maced. J. Chem. Chem. Eng.* (2010).

[65] Dueñas, M., Fernández, D., Hernández, T., Estrella, I. & Muñoz, R. Bioactive phenolic compounds of cowpeas (*Vigna**sinensis* L.). Modifications by fermentation with natural microflora and with *Lactobacillus**plantarum* ATCC 14917. *J. Sci. Food Agric.* **85**, 297–304. 10.1002/jsfa.1924 (2005).

[66] Hunlun, C., de Beer, D., Sigge, G. O. & Wyk, J. V. Characterisation of the flavonoid composition and total antioxidant capacity of juice from different citrus varieties from the Western Cape region. *J. Food Compos. Anal.* **62**, 115–125. 10.1016/j.jfca.2017.04.018 (2017).

[67] Killiny, N. Metabolomic comparative analysis of the phloem sap of curry leaf tree (*Bergera**koenegii*), orange jasmine (*Murraya**paniculata*), and Valencia sweet orange (*Citrus**sinensis*) supports their differential responses to Huanglongbing. *Plant Signal. Behav.* **11**, e1249080. 10.1080/15592324.2016.1249080 (2016). 27763819 10.1080/15592324.2016.1249080 PMC5157888

[68] Guimarães, R. et al. Targeting excessive free radicals with peels and juices of citrus fruits: Grapefruit, lemon, lime and orange. *Food Chem. Toxicol.* **48**, 99–106 (2010). 19770018 10.1016/j.fct.2009.09.022

[69] Alam, M. B. et al. High resolution mass spectroscopy-based secondary metabolite profiling of *Nymphaea**nouchali* (Burm. F.) stem attenuates oxidative stress via regulation of MAPK/Nrf2/HO-1/ROS pathway. *Antioxidants* **10**, 719 (2021). 34063678 10.3390/antiox10050719 PMC8147620

[70] Alshammari, F. et al. Profiling of secondary metabolites of optimized ripe Ajwa date pulp (*Phoenix**dactylifera* L.) using response surface methodology and artificial neural network. *Pharmaceuticals* **16**, 319 (2023). 37259461 10.3390/ph16020319 PMC9961821

[71] Abu-Reidah, I. M., del Mar Contreras, M., Arráez-Román, D., Fernández-Gutiérrez, A. & Segura-Carretero, A. UHPLC-ESI-QTOF-MS-based metabolic profiling of *Vicia**faba* L. (Fabaceae) seeds as a key strategy for characterization in foodomics. *Electrophoresis* **35**, 1571–1581 (2014). 24658881 10.1002/elps.201300646

[72] Priya Darsini, D. T., Maheshu, V., Vishnupriya, M., Nishaa, S. & Sasikumar, J. M. Antioxidant potential and amino acid analysis of underutilized tropical fruit *Limonia**acidissima* L. *Free Radic. Antioxid.* **3**, S62–S69. 10.1016/j.fra.2013.08.001 (2013).

[73] Aly, O. et al. Deciphering the potential of *Cymbopogon**citratus* (DC.) Stapf as an anti-obesity agent: Phytochemical profiling, in vivo evaluations and molecular docking studies. *Food Funct.* **15**, 12146–12168. 10.1039/D4FO04602A (2024). 39585680 10.1039/d4fo04602a

